# Introduction of ‘Generalized Genomic Signatures’ for the quantification of neighbour preferences leads to taxonomy- and functionality-based distinction among sequences

**DOI:** 10.1038/s41598-018-38157-3

**Published:** 2019-02-08

**Authors:** Konstantinos Apostolou-Karampelis, Dimitris Polychronopoulos, Yannis Almirantis

**Affiliations:** 1 Institute of Biosciences and Applications, National Center for Scientific Research “Demokritos”, 15310 Athens, Greece; 2 Genomics England, Charterhouse Square, London, EC1M 6BQ UK

## Abstract

Analysis of DNA composition at several length scales constitutes the bulk of many early studies aimed at unravelling the complexity of the organization and functionality of genomes. Dinucleotide relative abundances are considered an idiosyncratic feature of genomes, regarded as a ‘genomic signature’. Motivated by this finding, we introduce the ‘Generalized Genomic Signatures’ (GGSs), composed of over- and under-abundances of all oligonucleotides of a given length, thus filtering out compositional trends and neighbour preferences at any shorter range. Previous works on alignment-free genomic comparisons mostly rely on k-mer frequencies and not on distance-dependent neighbour preferences. Therein, nucleotide composition and proximity preferences are combined, while in the present work they are strictly separated, focusing uniquely on neighbour relationships. GGSs retain the potential or even outperform genomic signatures defined at the dinucleotide level in distinguishing between taxonomic subdivisions of bacteria, and can be more effectively implemented in microbial phylogenetic reconstruction. Moreover, we compare DNA sequences from the human genome corresponding to protein coding segments, conserved non-coding elements and non-functional DNA stretches. These classes of sequences have distinctive GGSs according to their genomic role and degree of conservation. Overall, GGSs constitute a trait characteristic of the evolutionary origin and functionality of different genomic segments.

## Introduction

In two pioneering works, Samuel Karlin and co-workers^1,2^ introduced the notion of the ‘genomic signature’, i.e. a vector composed by the ‘relative abundances’ (odds ratios) of dinucleotides. In this context, the nucleotide composition of the sequence had been filtered out by dividing the *observed frequency* of a given dinucleotide by its *expected frequency* (expected on the basis of mono-nucleotide composition). Namely, for any given dinucleotide XY, they used the ‘odds ratio’: ρ_XY_ = [XY]/([X][Y]), with X,Y ∈ {A,C,G,T}. Their work was along the lines with previous findings by Ruth Nussinov and Edward Trifonov^3,4^.

In a series of important publications^1,2,5–8^, Karlin and co-workers reported that genomic signature remains remarkably constant within a genome (contrary e.g. to GC-content which varies considerably within several eukaryotic genomes) while exhibiting inter-species variability. For comparison purposes they defined δ-distance as the rectilinear (Manhattan) distance between genomic signatures of any two sequences. They concluded that this distance can be utilized in phylogenetic reconstruction, as they found that higher δ-distances often imply species which are evolutionarily more distant than others with lower δ-distance. Motivated by such observations, in a recent work^9^ we used the genomic signatures for inferring the phylogenetic relationships among 340 bacteria, in comparison with several indices which account for compositional strand asymmetries. Not surprisingly, genomic signatures performed quite well, as the comparisons there were inter-species. Moreover, bacterial chromosomes are sufficiently long thus resulting to robust values due to reduced finite-size effects.

Due to the presumed intraspecies stability of the genomic signature, it seems that no one has systematically addressed the potential of δ-distance in distinguishing between sequence stretches of different functionality within the same genome. Such stretches with distinct neighbour preferences might be protein-coding regions versus Conserved Non-coding Elements (CNEs). CNEs are non-coding genomic regions highly conserved between two or more genomes, with important roles in early development^10,11^. In two previous studies^12,13^, we introduced novel machine-learning methodologies in order to distinguish among Conserved Non-coding Elements (CNEs)^14,15^, protein-coding exons and corresponding ‘surrogate sequences’ picked at random from the same genome. Additionally, we harnessed the power of δ-distance of genomic signatures to distinguish between the above classes of sequences as a baseline comparison. We observed that δ-distances performed relatively well, although they were computed for sequences originating from the same genome. This distinction apparently reflects the different functionalities of the examined sequences.

In the present work we explore a generalization of the concept of genomic signature to n-letter words, with n = 2, 3, 4 and 5. We employ a large set of bacterial genomes in order to assess the ability of these ‘*Generalized Genomics Signatures’* (GGSs) in deducing phylogenetic relationships. To this end, we make use of GGSs as input for machine learning classifiers and for reconstruction of the corresponding cladograms. Then, turning to the human genome, we consider concatenates of: (i) protein coding segments, (ii) conserved non-coding elements, and (iii) non-constrained sequence stretches picked at random (surrogate sequences). We perform clustering using the GGSs as feature vectors in order to assess their potential in intragenomic comparisons among sequences of different functionality.

## Methods

### Description of various datasets

#### Bacterial collection

We retrieved all bacterial genomes deposited in the NCBI database, available at the end of 2015. We retain only those genomes which belong to phyla with more than 10 members. Overall, our dataset is comprised of 2484 bacterial genomes. Note that among them, the genomes of 124 bacteria consist of multiple chromosomes.

#### CNE, exon and surrogate datasets taken from the human genome

*CNE 75–80*, *CNE 80–85*, *CNE 85–90* and *CNE 90–95* are CNE datasets composed *via* pairwise whole-genome comparisons between human and chicken (mapped on the human genome), as described in a previous work^12^. Together with *CNE 95–100* (previously named *UCNEs*^16^), they form a collection of CNE datasets derived from the same pairwise whole-genome comparisons, their only difference being the progressively increasing thresholds of conservation (from 75–80 to 95–100). *Mammalian CNEs* are sequences that are conserved within mammals but not found in chicken or fish, while *Amniotic CNEs* are conserved in mammals and chicken but not found in fish^17^. LiftOver^18^ is used in order to convert genomic coordinates from hg17 to hg19 release of the human genome. Mammalian CNEs are less constrained than any other CNE dataset used herein, as they are the most recently exapted CNEs, and are selected with a relatively low conservation threshold. For clustering purposes, Mammalian CNEs are split into two datasets of equal size, since they largely outnumber the other CNE collections. For the same purpose, the *exons of protein-coding genes*, collected as previously described^12^, are split into three equal size datasets. After splitting, Mammalian and exon datasets are of an approximate length of 10 Mnt (millions of nucleotides) each, while the length of the other CNE datasets range from 2.5 to 4.9 Mnt (full quantitative details are included in the *Supplementary information*). This is an additional reason for dividing the former classes of elements, in order to bring the sizes of the resulting datasets closer to the sizes of the latter ones.

Then, for each of these eleven datasets of constrained or functional elements (CNEs and exons) we compose one surrogate dataset in the following way: for each of the elements of every such dataset we pick and concatenate into the corresponding surrogate dataset a segment of equal length taken at random from the non-constrained, non-coding, repeat-masked human genome (release hg19).

These last concatenates may be seen either as *surrogates* of the corresponding constrained or protein-coding datasets, or as *a third class of sequences representing the non-repeated non-coding part of the genome* (see *Results and Discussion*).

### Generalized genomic signatures and δ-distances

Karlin and co-workers first introduced genomic signatures at the level of dinucleotides, as mentioned in the *Introduction*. Motivated by this concept, we present an extended version of genomic signatures for oligonucleotides of variable length. Let f(s) be the observed frequency (occurrence) of an n-letter word s (n-word) for any value of n: N_1_N_2_… N_n-1_N_n_ within a sequence S. For the computation of the *expected frequency* of s, E(s), we use the formula:

$${\rm{E}}({\rm{s}})={\rm{f}}({{\rm{N}}}_{1}{{\rm{N}}}_{2}\ldots {{\rm{N}}}_{{\rm{n}}-2}{{\rm{N}}}_{{\rm{n}}-1})\cdot {\rm{f}}({{\rm{N}}}_{2}{{\rm{N}}}_{3}\ldots {{\rm{N}}}_{{\rm{n}}-1}{{\rm{N}}}_{{\rm{n}}})/{\rm{f}}({{\rm{N}}}_{2}{{\rm{N}}}_{3}\ldots {{\rm{N}}}_{{\rm{n}}-2}{{\rm{N}}}_{{\rm{n}}-1}),$$

where f(N_1_N_2_… N_n-2_N_n-1_), f(N_2_N_3_… N_n−1_N_n_) and f(N_2_N_3_… N_n−2_N_n−1_) are the *observed frequencies* of the corresponding *n*-1 and *n*-2 sub-words within S. This method filters out compositional biases and has initially been introduced by Trifonov and co-workers^4^. Then, in order to measure the deviation of the observed frequency of the word s from its expected frequency in S, we use the ‘odds ratio’ ρ(s):

$${\rm{\rho }}({\rm{s}})={\rm{f}}({\rm{s}})/{\rm{E}}({\rm{s}})$$

As a first step in our analyses, we concatenate each sequence with its reverse complement, as suggested by Karlin *et al*.^2,5^, and then we proceed to further computations. This is done in order to incorporate into the computed quantities information about neighbour preferences in a strand-symmetric way. This becomes particularly important in the case of the study of DNA segments exhibiting strong inter-strand asymmetry. In such an artificial concatenate, reverse complementary oligo-nucleotides (n-words) have equal relative abundances by construction. Therefore, the ‘genomic signature’ of a given sequence as described by Karlin *et al*. should comprise the odds ratios of the four self-complementary dinucleotides (TA, AT, CG, GC) and twelve odds ratios, per two equal, corresponding to the pairs of the reverse complementary dinucleotides, e.g. CA·TG. Herein, for a given sequence S (concatenated with its reverse complement), we define as *Generalized Genomic Signature* (GGS) the vector containing the odds ratios for *all relevant n-words*, for any value of n. These *relevant n-words* include all self-complementary n-tuplets and only one from each pair of non self-complementary ones. For values of n which are odd numbers (n = 2k + 1, k ∈ ${\mathbb{N}}$) the GGS vector contains a number of elements ${w}_{n}=\frac{{4}^{n}}{2}={2}^{2n-1}$, because every vector element corresponds to an n-word and to its reverse complementary one. For values of n which are even numbers (n = 2k, k ∈ ${\mathbb{N}}$), each element of the GGS vector corresponds either to a self complementary (s.c.) n-word or to a pair of complementary (p.c.) n-words. Thus, in these cases ${w}_{n}={w}_{n}^{s.c.}+{w}_{n}^{p.c.}$. For self complementary words, each of their halves (of length k) determines the whole word completely, on the base of self complementarity. Thus, the number of self complementary words of length n = 2k equals the number of words of length k, namely ${w}_{n}^{s.c.}={4}^{k}={2}^{n}$. Consequently, the number of the remaining elements ${w}_{n}^{p.c.}$, which correspond to pairs of mutually complementary words is ${w}_{n}^{p.c.}=\frac{{2}^{2n}-{2}^{n}}{2}$, with 2^2n^ being the number of all n-words. Thereafter, for n being an even number, ${w}_{n}=\frac{{2}^{2n}-{2}^{n}}{2}+{2}^{n}={2}^{2n-1}+{2}^{n-1}$.

Hence, for n = 2, 3, 4 and 5, the numbers of elements of the corresponding GGS vectors are w_2_ = 10, w_3_ = 32, w_4_ = 136 and w_5_ = 512.

In the case of dinucleotides, the four self-complementary 2-words are: AT, TA, GC, CG and the six pairs of complementary 2-words are: AA·TT, AG·CT, AC·GT, CA·TG, GA·TC, CC·GG (ten vector elements in total). In Supplementary Table S1 the entire set of vectors considered in this work is included.

Karlin and co-authors introduced the rectilinear (Manhattan) distance of the vectors formed by all dinucleotides as a measure of the dissimilarity between two sequences g, h. We modify this concept for n = 2, 3, 4, 5 taking into account the elements of the GGS vectors as previously introduced. Hence, in the present study, for the dinucleotide genomic signature we set $\delta (g,h)=\frac{1}{{w}_{2}}\sum ^{XY}|{\rho }_{XY}(g)-{\rho }_{XY}(h)|$, X,Y ∈ {A,C,G,T}. Likewise, we compute the corresponding δ-distances for the higher order n-words. Note that in the initial formulation of the genomic signature^1^, Karlin and co-workers retained all 16 dinucleotide relative abundances, having however suggested that several alternative weighting schemes might be used in the formulation of the δ-distance. Nonetheless, in their following articles they always included all dinucleotides in δ-distance formulation, dividing by 16. Here, we chose to compose the generalized genomic signature vector, for any value of n, by including only one n-word from every pair of reverse complementary ones, along with all self-complementary n-words, should they exist, assigning equal weights to each of them.

### Classification of the bacterial dataset

For bacterial genomes with several chromosomes, we select the longest one. Thus, each bacterial species is represented by one chromosome. Overall, the dataset we use for classification consists of 2484 bacterial chromosomes. We partition our final collection into phyla, and we further divide Proteobacteria, which is the largest phylum, into classes (overall, 17 phyla and classes). For each bacterial species, we compute the GGS corresponding to *n*-words, for *n* = 2, 3, 4 and 5.

We perform classification analysis to assess whether GGSs suffice to predict the phylum or class in which bacterial species belong. To this end, we use the R package RWeka^19^, which interfaces to R the machine learning algorithms implemented in Weka. For each n = 2, 3, 4 or 5, we apply three classifiers to the corresponding set of GGSs. Namely, we use the J48, SMO and LMT classifiers. J48 generates C4.5 pruned decision trees using the Iterative Dichotomiser 3 algorithm^20^. SMO or *Sequential Minimal Optimization* is a time efficient algorithm for training support vector machines using polynomial or Radial Basis Function kernel (RBF kernels)^21^. LMT or *Logistic Model Trees* algorithm combines tree induction methods and logistic regression models, which are built at the leaves of the corresponding decision trees^20,22,23^. We assess the performance of each classifier by 10-cross validation experiments.

### Bacterial phylogeny reconstruction

Our dataset is comprised of all bacterial species considered for classification. As previously mentioned, we partition our collection into 17 major taxa (phyla and classes). Based on the *δ*-distances between GGS, we perform complete-linkage hierarchical clustering of the species belonging to the same phylum or class. Thus, we obtain the corresponding cladograms (GGS-based trees). In order to assess the performance of GGSs in reconstructing the phylogenetic relationships among bacterial species, we compare the topology of the GGS-based trees and the species trees that reflect the current consensus in systematic microbiology. More specifically, the species trees are retrieved from NCBI Taxonomy^24^, which provides a manually curated sequence-based phylogenetic classification. We perform the topological comparisons of GGS-based trees and species trees via the web-based tool Compare2Trees^25^. The resulting scores express the percent topological similarity of the trees in comparison.

## Results and Discussion

### Neighbour preferences implemented in the context of bacterial phylogeny reconstruction

#### Bacterial Classification

GGSs describe the correlations between consecutive nucleotides, from nearest-neighbouring (*n* = 2) to longer DNA strings (*n* = 3, 4, and 5). These correlations may reflect species-specific properties of DNA composition, as shown in previous studies^5,26^ in the case of dinucleotide relative abundances. This entails that the substitution rates of a given nucleotide depend on the identity of its neighbouring bases in a manner related to the evolutionary descent of each species. Such a relation might also be valid when we extend our analysis from nearest-neighbouring bases to more distant ones. The concept of GGSs provides us with the framework in which we can test this hypothesis.

**Figure 1:**
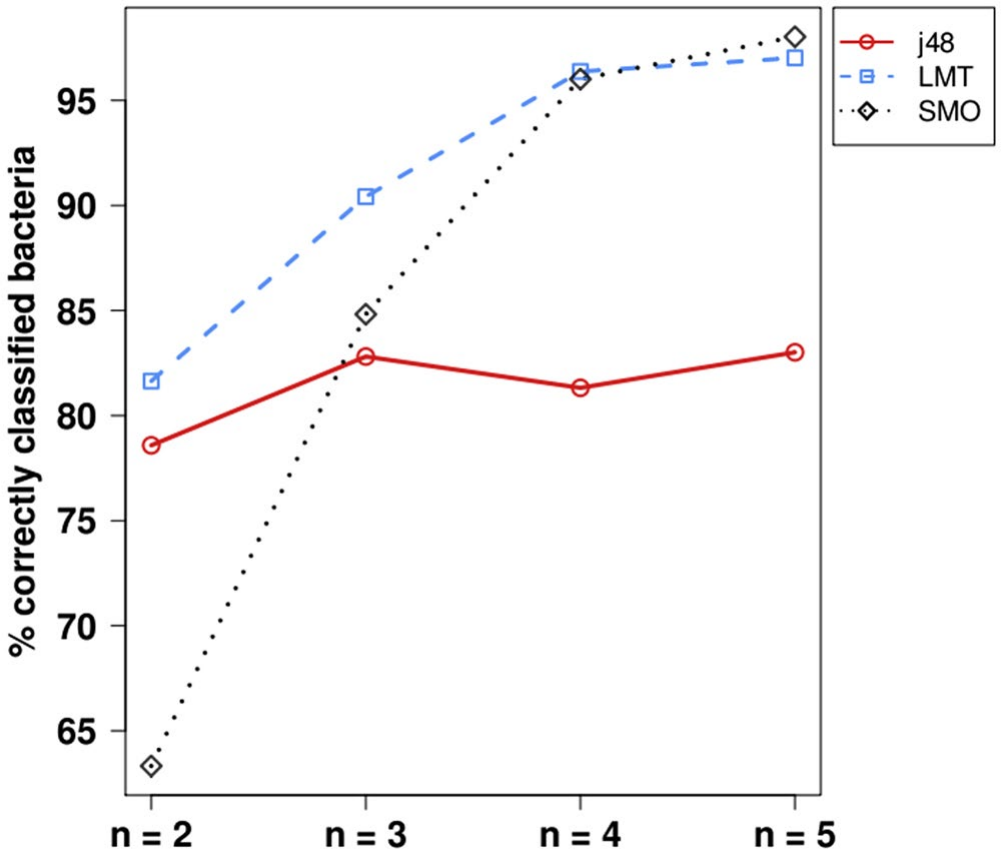
The percentage of correctly classified bacteria against the length *n* of the oligonucleotides used in the GGS analysis.

According to our definition of GGSs, we attribute to each bacterial genome of our collection a vector of relative abundances of all relevant *n*-words for *n* = 2, 3, 4 and 5. Then, we consider the elements of each vector as explanatory variables in a series of classification experiments that we perform in order to determine whether GGSs suffice to predict the phylum or class in which the corresponding bacterial species belong. We employ three classifiers, namely the J48 decision trees, the SMO implementation for support vector machines, and the Logistic Model Trees (LMT) algorithms. To evaluate our classifiers, we perform 10-fold cross validation and summarize our results in Table 1. The statistics we present are the weighted averages for all phyla or classes, since our experiments correspond to non binary classification problems. A concise presentation of our classification study is provided in Fig. 1, where the percentage of correctly classified bacteria is depicted against the length of the oligonucleotides which constitute the corresponding GGSs.

**Table 1:** Classification of bacteria based on Generalized Genomic Signatures.

	TP Rate	FP Rate	Precision	Recall	F-Measure	MCC	ROC Area
**j48**							
n = 2	0.786	0.031	0.787	0.786	0.786	0.756	0.895
n = 3	0.828	0.022	0.827	0.828	0.827	0.806	0.913
n = 4	0.813	0.024	0.812	0.813	0.811	0.789	0.904
n = 5	0.830	0.020	0.833	0.830	0.831	0.810	0.912
**SMO**							
n = 2	0.633	0.096	0.630	0.633	0.602	0.552	0.875
n = 3	0.848	0.029	0.846	0.848	0.841	0.822	0.961
n = 4	0.960	0.007	0.960	0.960	0.960	0.955	0.991
n = 5	0.980	0.003	0.980	0.980	0.980	0.978	0.995
**LMT**							
n = 2	0.816	0.030	0.817	0.816	0.815	0.789	0.938
n = 3	0.904	0.014	0.905	0.904	0.904	0.892	0.978
n = 4	0.964	0.005	0.964	0.964	0.964	0.959	0.996
n = 5	0.970	0.005	0.970	0.970	0.970	0.966	0.998

Weighted average statistics denoting the performance of the classifiers (J48, SMO, LMT) we applied to our dataset of GGSs, for n = 2, 3, 4 and 5. TP Rate: the rate in which species are correctly classified into each bacterial phylum/class (True Positive). FP Rate: the rate in which species are classified into bacterial phyla/classes in which they do not belong (False Positive). Precision: the fraction of species correctly classified into each bacterial phylum/class. Recall: the fraction of correctly classified species over the number of species that actually belong to each bacterial phylum/class. F-measure: weighted average of precision and recall (1: perfect accuracy, 0: no accuracy). MCC: Matthews correlation coefficient between the current consensus in bacterial phylogeny and the predicted classification; its values ranges from 1 to -1 (1: perfect prediction, 0: random classification, −1: total disagreement between predicted and consensus classification). ROC Area: The area under the Receiver Operating Characteristic curve, corresponding to the probability that a randomly selected species will be assigned to the correct bacterial phylum/class instead of another randomly selected species which does not belong to this bacterial phylum/class.

An overall inspection of Table 1 shows that GGSs can be used efficiently in order to classify bacteria in their corresponding major taxa. Values of ROC area range from 0.875 to 0.998 and illustrate the high performance of all tested classifiers. Moreover, as we shift from dinucleotides to longer n-words, all classification metrics significantly improve, with the only exception of the J48 classifier for n = 4 (see also Fig. 1). The increase of bacteria correctly classified to their corresponding phyla or classes is particularly evident when comparing the values of F-measure for 2- and 5-nucleotides (e.g. in the case of SMO, for *n* = 2 and *n* = 5, F-measure increases from 0.602 to 0.980). Once we take into account more distant nucleotides than first neighbours (n > 2), a stronger correlation is exhibited between the predicted classification and the consensus taxonomy of bacteria (e.g. in the case of LMT, for *n* = 2 and *n* = 5, MCC increases from 0.789 to 0.966). Our findings suggest that the substitution rates of a given nucleotide are shaped by a surrounding sequence which extends beyond first neighbours, in a way that reflects the major taxonomic divisions of bacteria. We present herein the results of all three classifiers we initially tested, in order to illustrate their unequal performance and facilitate their ranking. The reason for this is the well-known need to try several algorithms for any specific problem of classification in order to determine which is the optimal for this particular task. It has been shown that no optimal classifier exists independently of the problem to be addressed^27^, because a general-purpose, universal optimization strategy is impossible^28^.

#### Reconstruction of Bacterial Phylogeny

**Figure 2:**
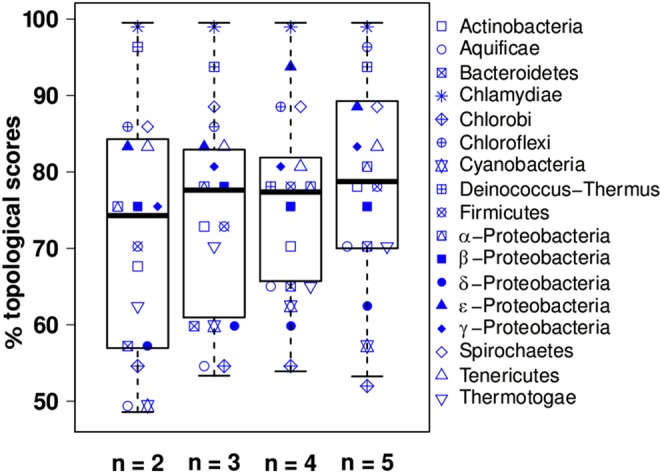
Boxplots of topological scores. For each phylum or class, the scores reflect the percent topological similarity of the GGS-based trees and consensus species trees. The higher the scores, the more accurately GGSs capture the phylogenetic relationships between bacteria. GGSs are calculated for n-words, with n = 2, 3, 4 and 5.

In the previous section we demonstrate that GGSs can be utilized to produce a coarse classification of bacterial species at the level of phylum or class. We further evaluate the performance of GGSs in tracing the phylogenetic relationships among bacteria within these major taxa. To this end, we use GGSs to construct cladograms of all species belonging to the same phylum or class and compare these GGS-based trees to the corresponding species trees (see *Methods*). The resulting topological scores are depicted via boxplots in Fig. 2.

For *n* = 2, the corresponding scores are rather scattered and the median equals 74,3%, while for *n* = 3 or 4 the scores distribution becomes more compact and the median is slightly increased. For n = 5, the resulting distribution of topological scores is shifted towards higher values, with a median equal to 78,7%.

Overall, for most taxonomic subdivisions we consider, the inferred topology of the GGS-based trees concurs with bacterial phylogeny. Moreover, and in accordance with our classification findings, GGSs incorporate more phylogenetic information as we shift from *n* = 2 to *n* = 5. Thus, the mutual dependence of distant nucleotides, as described by GGSs for *n* > 2, exhibits a stronger correlation with the evolutionary descent of bacteria compared to their nearest-neighbour dependencies that are hitherto utilized for phylogenetic inference.

Previous studies on alignment-free genomic comparisons (for comprehensive reviews see^29–31^) employ the observed k-mer frequencies without decoupling the neighbour preferences from the background nucleotide composition. On the contrary, our analysis based on GGSs quantifies only the neighbour preferences at a specific distance (n value) in the relevant GGS vector. Thus, direct comparison of the performance of these two approaches (k-mer based and GGS based) can be misleading. Having that in mind we repeat the analysis we hitherto performed, using observed k-mer frequencies instead of odds ratios for the different values of n (or k).

In the *Supplementary information* we present the results of this analysis in the form of Supplementary Table S2 and Supplementary Fig. S1, corresponding to Table 1 and Fig. 2, respectively. We find that observed frequencies of k-mers perform equally or slightly better than odds ratios (expressing neighbour preferences) in correctly assigning genomes to the major phyla or classes (see Table 1 vs. Supplementary Table S2). On the other hand, neighbour preferences, as expressed by odds ratios, perform better than the observed k-mer frequencies in phylogenetic reconstruction within phyla or classes (see Fig. 2 vs. Supplementary Fig. S1). This finding can be understood if we take into account that major taxonomic divisions often significantly differ with regard to their nucleotide composition, with most prominent their variability in GC content. Composition supplemented with neighbour preferences (both encoded in k-mer analysis) lead to the preponderance of k-mers in detecting the phylum where a genome belongs. On the other hand, taxonomically closer genomes are less divergent from the point of view of nucleotide composition. Thus, fractionated (for different n values) neighbour preferences can be applied more efficiently in phylogenetic reconstruction. The above comparisons show the clear difference between k-mer based and neighbour preferences based analyses, in both the type of the used quantities and the related research questions which can be addressed using any of these two approaches.

As mentioned by Karlin and Burge^5^, environmental conditions such as temperature influence bacterial taxa not only in their nucleotide composition (e.g. higher GC content at higher temperatures), but also in dinucleotide preferences. More recent studies reveal that the lifestyle of bacteria greatly affects their proteome, shaping discrete temperature-dependent profiles of amino acid composition^32^. Our findings on bacterial GGSs clearly show that even when we filter out nucleotide composition, the remaining evolutionary traces of discrete lifestyles on the genomic neighbour preferences are still able to serve purposes of classification.

#### Intra- and inter-genome comparisons

In what follows the considered dataset comprises the unique chromosome of most bacterial genomes (2360 out of 2484) of our collection and the two lengthier chromosomes of the 124 species with more than one chromosome. Plasmids are excluded from our analysis. Employing *δ*-distances, we pairwise compare the corresponding GGSs for *n* = 2, 3, 4 and 5. We then sort in ascending order all pairs of chromosomes. Thus, for a given pair of chromosomes, the higher its rank along the resulting array is, the more similar the corresponding GGSs are. For *n* = 2, 3, 4 and 5, we detect along our sorted arrays the *rank of similarity* of chromosomes belonging to the same bacterial genome.

**Figure 3:**
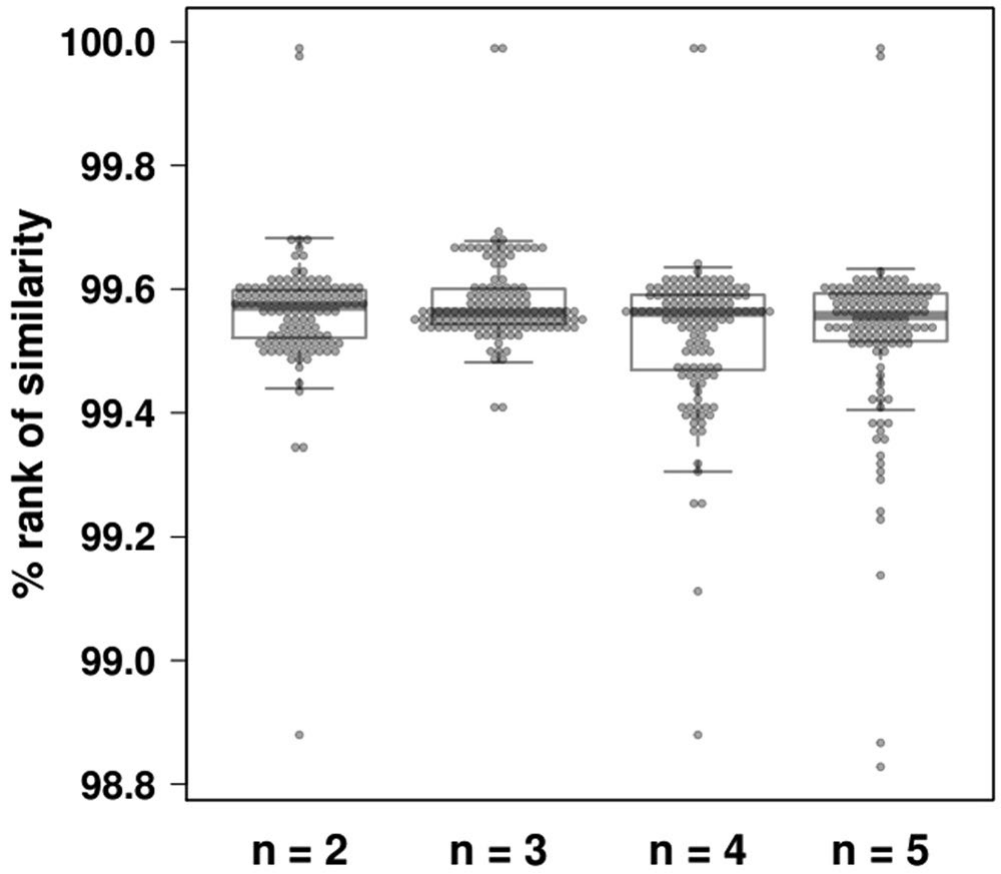
Boxplots of percent *rank of similarity* of chromosome pairs belonging to the same genome. The higher the rank is, the more similar the corresponding GGSs are. For details, see text.

Figure 3 represents the percent *rank of similarity*, in terms of GGSs, between chromosomes belonging to the same genome. The plotted ranks range from a minimum of 98.83%, for *n* = 5, up to a maximum of 99.99%, for any value of *n*, while the corresponding median values are equal up to the first decimal place (99.6%). Overall, different chromosomes of the same genome have very similar GGSs compared to chromosomes belonging to different species. Taking into account this analysis along with the phylogenetic reconstruction we presented in the previous section, it can be argued that GGSs exhibit strong intra-genome stability while at the same time their inter-genome variability suffice to distinguish between different bacteria. The observed intragenomic homogeneity in terms of GGSs indicates that the systematic dependency of substitution rates on the wide region in which a given nucleotide lies remains constant when assessed at a genome-wide scale in bacterial species.

### Clustering experiments involving eukaryotic constrained and non-constrained sequences

In the *Supplementary Information* we present a naïve classification scheme of our eukaryotic datasets (sequence concatenates of human origin). Namely, we perform pairwise comparisons based on δ-distances, in order to assess whether the GGS vectors cluster according to the specific functionality of the corresponding datasets. We found that in almost all cases δ-distances of datasets of the same type (CNEs, exons or sequences representative of the bulk of the non-repetitive genome) are shorter than distances of datasets of two different types. Thus, all pairs of sequence concatenates are sharply divided into two groups, with ‘short’ or ‘long’ distances, for datasets of similar or different functionality respectively. Apart from only one, the few sporadic violations of this observation involve the comparison between a dataset of ‘Mammalian CNEs’ and another CNE dataset, which are located in the ‘long distances’ group. As mentioned in the *Methods* section, Mammalian CNEs are selected under the less strict conditions of sequence conservation. Thus, the aforementioned pairs of datasets, although both CNEs, are indeed expected to exhibit relatively long distances, as the Mammalian CNEs abide less than all other CNEs to the conjectured compositional similarity of this class of sequences. This simple approach has the particularity to be independent of any elaborate feature of the existing clustering algorithms. Nonetheless the obtained result converges with the findings of other types of clustering we present in the following sections.

#### Visualisation of clustering through principal component analysis

**Figure 4:**
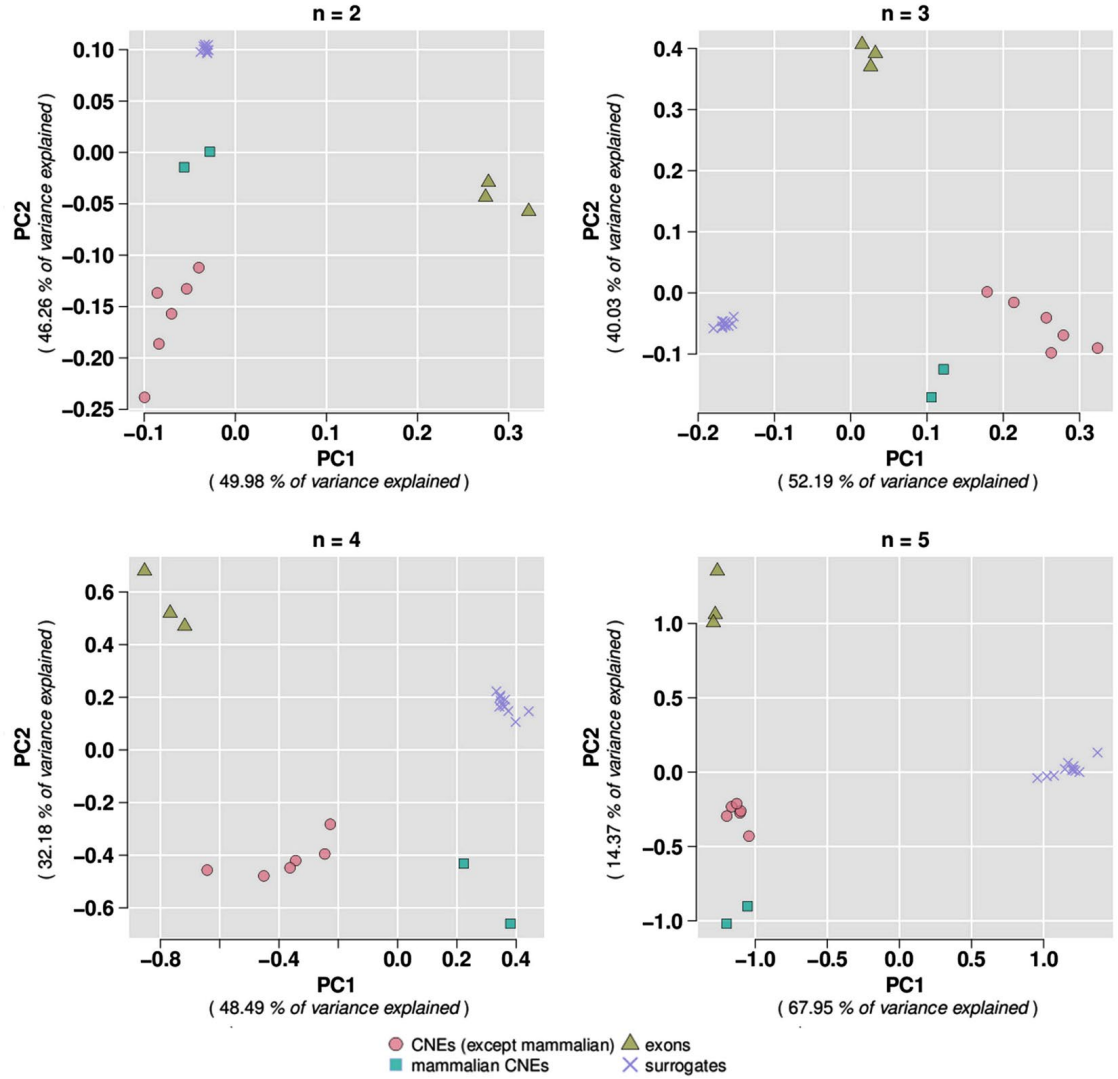
PCA plots corresponding to GGS vectors for n = 2, 3, 4 and 5. CNEs, exons and surrogate sequences are largely grouped together. For details, see text.

We perform Principal Component Analysis (PCA) using the 22 datasets derived from the human genome. We plot the first two principal components corresponding to GGS vectors for n = 2, 3, 4 and 5. PCA plots reveal a clear clustering of the studied datasets, as shown in Fig. 4. Exons and surrogates do form distinct groups, while in the case of Conserved Non-coding Elements (CNEs) we observe a further subdivision; namely, six categories of CNEs fall within the same cluster while Mammalian CNEs stand out. Interestingly, five of the aforementioned six CNE datasets are CNEs conserved between human and chicken which have been identified using the same method but with progressively increasing conservation thresholds (CNEs 75–80, 80–85, 85–90, 90–95, 95–100, see *Methods* and a previous study^12^). The sixth dataset of this cluster is Amniotic CNEs which contains elements also conserved in chicken. On the other hand, Mammalian CNEs are the least conserved and the most recently exapted elements. Consequently, they represent an ancestral genome which is more recent than the one represented by the Amniotic and the other considered datasets. This difference in the degree of conservation is effectively captured by the PCA analysis, according to which Mammalian CNEs form a clearly outlying group. Amniotic and Mammalian CNE datasets have been identified in the same study^17^ and it is noteworthy that the GGS methodology manages to capture their different evolutionary depths.

In line with the above argument, in Table 2 we present δ-distances of the five CNE datasets with their corresponding surrogates, which form an ordered series of increasing degree of conservation, for n = 2, 3, 4 and 5. We observe that the δ-distance of these CNE datasets from their corresponding surrogates follows the increasing conservation for all values of n without exception. This is a clear indication of the preservation of significant information about ancestry and function within the sequence composition of CNEs as quantified through the GGS methodology. Here again, we ascertain that not only dinucleotides but lengthier oligonucleotides also do contain function- and ancestry-related information expressed in their relative abundances. Note that δ-distances between n-words of different lengths are not comparable, since their magnitudes differ by construction.

**Table 2:** δ-distances between CNE datasets and the corresponding surrogates.

	n = 2	n = 3	n = 4	n = 5
CNE 75–80	0.5021	1.5158	6.9035	39.2900
CNE 80–85	0.5342	1.6710	7.3115	40.6388
CNE 85–90	0.5907	1.9277	8.2785	41.6063
CNE 90–95	0.6470	2.0067	9.5598	43.4783
CNE 95–100	0.7183	2.2325	10.5329	46.7692

CNE datasets conserved between Homo and chicken for increasing percent thresholds of conservation. δ-distances are measured between each CNE dataset and the corresponding surrogates composed of DNA stretches picked at random from the non-conserved, non-repeated bulk of the genome.

#### K-means clustering reveals the biological significance of GGSs in distinguishing between different functionalities

**Figure 5:**
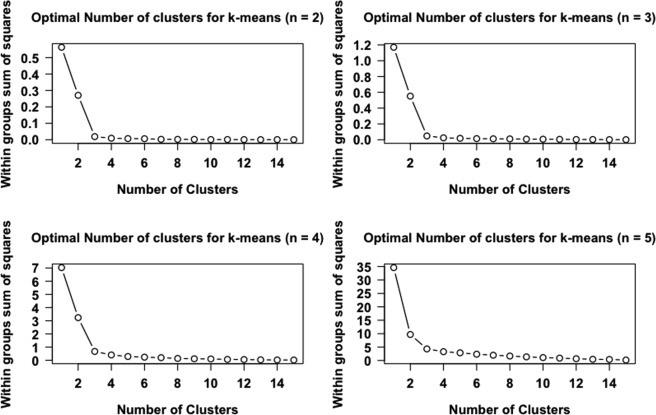
The ‘elbow’ method determines the optimal number of clusters for the k-means clustering of GGSs computed for CNE, exons and surrogate datasets, excluding mammalian CNEs. For the computation of GGSs, word length takes values from 2 to 5.

We proceed to k-means clustering using GGS feature vectors as input, computed for the aforementioned datasets. We use the ‘elbow’ method to decide on the optimal number of clusters for the k-means^33^. The elbow method provides a way of interpretation and validation of consistency within cluster analysis designed to assist in finding the appropriate number of clusters in a dataset. In brief, we run k-means clustering of the CNE, exon and surrogate surrogates (for the corresponding values of n), for a range of values of k (from 1 to 15). Maximum number of iterations is set to 50 and twenty random datasets are chosen for each iteration. For each value of k, we calculate the within groups sum of squares (WSS). Then, we plot a line chart of the WSS for each value of k. If the line chart resembles an arm, then the ‘elbow’ on the arm is the value of k that is the optimal number of formed clusters. We first omit the Mammalian CNE dataset since they are considered to be outliers, as evidenced from the ‘naïve classification scheme’ and the PCA (Fig. 4) described above. Indeed, excluding Mammalian CNEs leads to the formation of three clusters (k = 3), which correspond to CNEs, exons and surrogates respectively, for all values of n (Fig. 5). When we consider all datasets, we observe that the value of k where the ‘elbow’ is formed is clearly 4 (see Supplementary Fig. S2). This is compatible with the different ancestry of the Mammalian CNE dataset as also commented above. Taken together, our findings demonstrate the potential of the method we proposed to distinguish between constrained and non-constrained sequences of eukaryotic genomes.

### Concluding remarks and perspectives

The present work investigates the correlation between genomic composition, as expressed by the Generalized Genomic Signatures (GGSs) defined herein, and genomic function or phylogeny. There is no general evidence that this correlation expresses direct causal relationships in all cases. Perhaps, in several instances, it reflects a common cause behind both the oligonucleotide relative abundances and the correlated feature. The proposed GGS methodology is able to fractionate genomic composition at the levels of first, second, third, etc neighbours. It is worth emphasizing that the performance of GGS for n^th^ order neighbours is not merely an improvement of GGS for n-1, in phylogenetic reconstruction or in the correct prediction of function intragenomically. GGSs for different values of n represent independent features of genomes or sequences, contrary to k-mers, which incorporate information of all their corresponding subwords and are extensively used in alignment-free sequence comparisons. For instance, k-mers for k = 3 contain information about nucleotide composition, first neighbours’ preferences, plus information about second neighbours. In the present study, the consecutive levels of neighbourhood information are decoupled. Thus, it remains non trivial if we find that GGSs for different values of n perform in a comparable way in taxonomic reconstruction or distinguishing among genomic elements, as the neighbour preferences encoded in GGSs of higher n values does not include information about lower distances. Here, analysis for each value of n brings new information about neighbour preferences at consecutive distances. Alignment-free methods for sequence analysis are often, but not exclusively, based on k-mer frequencies. For early studies in this field Vinga and Almeida^29^ wrote a very informative review, and in the subsequent years much research appeared investigating alignment-free methods. For more recent comprehensive reviews see^30,31^. However, the dependence of observed n-tuplets’ frequencies on the frequencies of their sub-sequences (sub-words) has been only sparsely addressed in the relevant literature^34^. To the best of our knowledge, no work has developed a metrics of distance between sequences uniquely based on neighbour preferences at a given length n, as we attempt here. Moreover, it is interesting that the three reviews on alignment free methods we mentioned above, very informative from many points of view, give no reference at all to the work of Karlin and his group on ‘genomic signatures’, although they use the term with a different meaning.

The presented results show an unexpectedly strong correlation between several functional or evolutionary traits and relative abundances, not only of dinucleotides but also of longer oligonucleotides (n-words). This might indicate that at least part of the overall genomic dynamics (including protein-DNA and other relevant molecular interactions) is conditioned by a relatively extended nucleotide region surrounding the position on the sequence which is traditionally considered to be the focus of a relevant molecular recognition event. For instance, and in the case of CNEs, literature suggests that overlapping Transcription Factor Binding Sites (TFBS) are usually found within those constrained elements^35,36^. However, there is still no clear demonstration that overlapping binding sites would suffice to explain the extreme non-coding conservation observed in CNEs^14^.

All the principal findings presented herein can be understood as consequences of the constraints shaping different genomes or parts of a genome with different functionalities. As Karlin and his associates have argued, first neighbour preferences mainly reflect mutational dynamics. Our contribution indicates that this may be extended to further located neighbours too. The parallels between the consensus bacterial ancestry and cladograms constructed on the basis of GGSs advocate in favour of the robustness of this result. In the case of the human genome, GGSs are able to clearly distinguish among the unconstrained bulk of the genome, protein coding sequences and CNEs. The composition of non-constrained, non-repetitive sequences mostly represents the compositional profile imposed by mutational rates. On the other hand, the composition of the protein-coding sub-genome, as expressed by GGSs, is expected to be shaped by a combination of mutational pressure characteristic of the specific genome and the average compositional constraints imposed by the amino acid content of the proteome. Furthermore, CNEs might be seen as representative of the composition of ancestral genomes; mostly, of the last common ancestor of the present-day genomes used for the identification of the considered CNE dataset. This view explains the gradual increase of the δ-distance of a series of CNE collections from their surrogate sequences, when these CNE datasets are produced by gradual increase of the conservation thresholds (see Table 2). Moreover, the high performance of GGSs in distinguishing among all examined CNE classes, for all considered lengths *n*, does not support the suggestion that the selective pressure shaping CNEs, and especially the more extremely conserved ones (mentioned as Ultra-Conserved Elements or UCEs in that work), is exerted on the single nucleotide level^37^, since GGSs capture nucleotide correlations extending along whole stretches of DNA.

We introduce Generalised Genomic Signatures and their corresponding δ-distances as a generalization of the initial construction of Karlin and co-workers. The results we present herein demonstrate the possible applications of the proposed concept of GGSs to further elucidate current questions in genomic studies. In what follows we indicatively mention some of the topics which can be further investigated by using the framework of our analysis.

A future systematic study might include several genomic elements, like e.g. gene promoters or enhancers, for which it is unknown whether there is an interplay between functionality and sequence composition across their entire length. We expect that such regions might have characteristic (generalized genomic) signatures with relatively high δ-distances from the signature of the bulk of the genome, thus forming distinct clusters according to their functionality. More generally, exploiting present knowledge of genomic structure, the tool of GGSs could be used in order to investigate the role of what E. Zuckerkandl had named ‘polite DNA’ in an early work. This term has been used to characterise any portion of a genome that “without being crucially involved in function, is subject to constraints of conformity and, through its base composition, respects a function for which it is not (absolutely) required”^38^.

Using the methodology we apply for assessing the similarity between GGSs-based cladogram and the consensus phylogenetic trees of bacteria, an analogous investigation could be undertaken for a collection of eukaryotic genomes representative of major animal taxa. Given the sharp distinction we detect in eukaryotes among GGSs according to sequence functionality, phylogenetic reconstruction can be performed using either protein coding or non-repetitive, non-coding DNA. Comparisons of these results might lead to interesting conclusions about similarities or divergences in neighbour preferences between protein-coding and non-constrained parts of the genome. Moreover, motivated by the results of Fig. 3, several chromosomes from each eukaryote might be used in order to test for inter- vs. intra-genomic variations of compositional profiles.

## Supplementary information

Supplementary Information
