# Supplementary material for: Introduction of ‘Generalized Genomic Signatures’ for the quantification of neighbour preferences leads to taxonomy- and functionality-based distinction among sequences
Source: Sci Rep. 2019 Feb 8;9:1700. doi: 10.1038/s41598-018-38157-3 (PMC6368578; doi:10.1038/s41598-018-38157-3)
Supplement: Supplementary file 1 — Supplementary Information [file 41598_2018_38157_MOESM1_ESM.pdf]

# Introduction of ‘Generalized Genomic Signatures’ for the quantification of neighbour preferences leads to taxonomy- and functionality-based distinction among sequences

## Supplementary information

**Konstantinos Apostolou-Karampelis[1,†], Dimitris Polychronopoulos[2,†], and Yannis Almirantis[1,*]**

[1]Institute of Biosciences and Applications, National Center for Scientific Research “Demokritos”, 15310 Athens, Greece

[2]Genomics England, Charterhouse Square, London EC1M 6BQ, UK

[†]These authors contributed equally to the paper as first authors

[*]To whom correspondence should be addressed. Tel: +302106503601. Email: yalmir@bio.demokritos.gr

## Contents


| Subject | page |
|---|---|
| Supplementary Table S1 [The full set of GGS elements for n = 2-5] | 3 |
| k-mer based genome analysis | 7 |
| Supplementary Table S2 | 7 |
| Supplementary Figure S1 | 8 |
| CNE, exon and surrogate datasets | 9 |
| Naïve search for clustering of eukaryotic sequences with different functionalities | 10 |
| Duplets | 11 |
| Triplets | 16 |
| Tetraplets | 21 |
| Pentaplets | 26 |
| Supplementary Figure S2 [Graphical evaluation of optimal number of clusters for the complete set of human-origin datasets including 'mammalian CNEs'] | 32 |

# The full set of GGS elements for $n$ = 2,3,4,5

## $n$ = 2

| | | | | | |
|---|---|---|---|---|---|
| AA (TT) | AG (CT) | AC (GT) | CA (TG) | GA (TC) | CC (GG) |

***self complementary***

| | | | |
|---|---|---|---|
| AT | TA | CG | GC |

## $n$ = 3

| | | | |
|---|---|---|---|
| AAA (TTT) | ACT (AGT) | TTC (GAA) | CAG (CTG) |
| AAT (ATT) | ACC (GGT) | TTG (CAA) | CTC (GAG) |
| AAC (GTT) | ACG (CGT) | TCA (TGA) | CCC (GGG) |
| AAG (CTT) | AGA (TCT) | TCC (GGA) | CCG (CGG) |
| ATA (TAT) | AGG (CCT) | TCG (CGA) | CGC (GCG) |
| ATC (GAT) | TAA (TTA) | TGC (GCA) | GAC (GTC) |
| ATG (CAT) | TAC (GTA) | TGG (CCA) | GCT (AGC) |
| ACA (TGT) | TAG (CTA) | CAC (GTG) | GCC (GGC) |

## $n$ = 4

| | | | | | | | |
|---|---|---|---|---|---|---|---|
| AAAA (TTTT) | ATAA (TTAT) | AGAG (CTCT) | ACTA (TAGT) | TAGG (CCTA) | TGAG (CTCA) | TCCG (CGGA) | GGAG (CTCC) |
| AAAT (ATTT) | ATAG (CTAT) | AGAC (GTCT) | ACTG (CAGT) | TAGC (GCTA) | TGAC (GTCA) | TCCC (GGGA) | GGTG (CACC) |
| AAAG (CTTT) | ATAC (GTAT) | AGTA (TACT) | ACTC (GAGT) | TACA (TGTA) | TGTG (CACA) | GAAG (CTTC) | GGGG (CCCC) |
| AAAC (GTTT) | ATTA (TAAT) | AGTG (CACT) | ACGA (TCGT) | TACG (CGTA) | TGTC (GACA) | GAAC (GTTC) | GGGC (GCCC) |
| AATA (TATT) | ATTG (CAAT) | AGTC (GACT) | ACGG (CCGT) | TACC (GGTA) | TGGA (TCCA) | GATG (CATC) | GGCG (CGCC) |
| AATG (CATT) | ATTC (GAAT) | AGGA (TCCT) | ACGC (GCGT) | TTAG (CTAA) | TGGG (CCCA) | GAGG (CCTC) | GCAG (CTGC) |
| AATC (GATT) | ATGA (TCAT) | AGGT (ACCT) | ACCA (TGGT) | TTAC (GTAA) | TGGC (GCCA) | GAGC (GCTC) | GCTG (CAGC) |
| AAGA (TCTT) | ATGT (ACAT) | AGGG (CCCT) | ACCG (CGGT) | TTTG (CAAA) | TGCG (CGCA) | GACG (CGTC) | GCGG (CCGC) |
| AAGT (ACTT) | ATGG (CCAT) | AGGC (GCCT) | ACCC (GGGT) | TTTC (GAAA) | TGCC (GGCA) | GACC (GGTC) | GCCG (CGGC) |
| AAGG (CCTT) | ATGC (GCAT) | AGCA (TGCT) | TAAA (TTTA) | TTGA (TCAA) | TCAG (CTGA) | GTAG (CTAC) | CAAG (CTTG) |
| AAGC (GCTT) | ATCA (TGAT) | AGCG (CGCT) | TAAG (CTTA) | TTGG (CCAA) | TCAC (GTGA) | GTTG (CAAC) | CAGG (CCTG) |
| AACA (TGTT) | ATCT (AGAT) | AGCC (GGCT) | TAAC (GTTA) | TTGC (GCAA) | TCTG (CAGA) | GTGG (CCAC) | CACG (CGTG) |
| AACT (AGTT) | ATCG (CGAT) | ACAA (TTGT) | TATG (CATA) | TTCA (TGAA) | TCTC (GAGA) | GTGC (GCAC) | CTGG (CCAG) |

| | | | | | | | |
|---|---|---|---|---|---|---|---|
| AACG<br>(CGTT) | ATCC<br>(GGAT) | ACAG<br>(CTGT) | TATC<br>(GATA) | TTCG<br>(CGAA) | TCGG<br>(CCGA) | GTCG<br>(CGAC) | CTCG<br>(CGAG) |
| AACC<br>(GGTT) | AGAA<br>(TTCT) | ACAC<br>(GTGT) | TAGA<br>(TCTA) | TTCC<br>(GGAA) | TCGC<br>(GCGA) | GTCC<br>(GGAC) | CGGG<br>(CCCG) |
| ***self complementary*** | | | | | | | |
| AATT | AGCT | TATA | TGCA | GATC | GGCC | CATG | CGCG |
| ATAT | ACGT | TTAA | TCGA | GTAC | GCGC | CTAG | CCGG |

## ***n* = 5**

| | | | | | | | |
|---|---|---|---|---|---|---|---|
| AAAAA<br>(TTTTT) | ATAAG<br>(CTTAT) | AGAGG<br>(CCTCT) | ACTTC<br>(GAAGT) | TAGCA<br>(TGCTA) | TGACG<br>(CGTCA) | GAAAG<br>(CTTTC) | GGTGG<br>(CCACC) |
| AAAAT<br>(ATTTT) | ATAAC<br>(GTTAT) | AGAGC<br>(GCTCT) | ACTGA<br>(TCAGT) | TAGCG<br>(CGCTA) | TGACC<br>(GGTCA) | GAAAC<br>(GTTTC) | GGTGC<br>(GCACC) |
| AAAAG<br>(CTTTT) | ATATA<br>(TATAT) | AGACA<br>(TGTCT) | ACTGG<br>(CCAGT) | TAGCC<br>(GGCTA) | TGTAG<br>(CTACA) | GAATG<br>(CATTC) | GGTCG<br>(CGACC) |
| AAAAC<br>(GTTTT) | ATATG<br>(CATAT) | AGACT<br>(AGTCT) | ACTGC<br>(GCAGT) | TACAA<br>(TTGTA) | TGTAC<br>(GTACA) | GAATC<br>(GATTC) | GGGAG<br>(CTCCC) |
| AAATA<br>(TATTT) | ATATC<br>(GATAT) | AGACG<br>(CGTCT) | ACTCA<br>(TGAGT) | TACAG<br>(CTGTA) | TGTTG<br>(CAACA) | GAAGG<br>(CCTTC) | GGGTG<br>(CACCC) |
| AAATT<br>(AATTT) | ATAGA<br>(TCTAT) | AGACC<br>(GGTCT) | ACTCG<br>(CGAGT) | TACAC<br>(GTGTA) | TGTTC<br>(GAACA) | GAAGC<br>(GCTTC) | GGGGG<br>(CCCCC) |
| AAATG<br>(CATTT) | ATAGT<br>(ACTAT) | AGTAA<br>(TTACT) | ACTCC<br>(GGAGT) | TACTG<br>(CAGTA) | TGTGA<br>(TCACA) | GAACG<br>(CGTTC) | GGGGC<br>(GCCCC) |
| AAATC<br>(GATTT) | ATAGG<br>(CCTAT) | AGTAG<br>(CTACT) | ACGAA<br>(TTCGT) | TACTC<br>(GAGTA) | TGTGG<br>(CCACA) | GAACC<br>(GGTTC) | GGGCG<br>(CGCCC) |
| AAAGA<br>(TCTTT) | ATAGC<br>(GCTAT) | AGTAC<br>(GTACT) | ACGAG<br>(CTCGT) | TACGA<br>(TCGTA) | TGTGC<br>(GCACA) | GATAG<br>(CTATC) | GGGCC<br>(GGCCC) |
| AAAGT<br>(ACTTT) | ATACA<br>(TGTAT) | AGTTA<br>(TAACT) | ACGAC<br>(GTCGT) | TACGG<br>(CCGTA) | TGTCG<br>(CGACA) | GATAC<br>(GTATC) | GGCAG<br>(CTGCC) |
| AAAGG<br>(CCTTT) | ATACT<br>(AGTAT) | AGTTG<br>(CAACT) | ACGTA<br>(TACGT) | TACGC<br>(GCGTA) | TGTCC<br>(GGACA) | GATTG<br>(CAATC) | GGCTG<br>(CAGCC) |
| AAAGC<br>(GCTTT) | ATACG<br>(CGTAT) | AGTTC<br>(GAACT) | ACGTG<br>(CACGT) | TACCA<br>(TGGTA) | TGGAG<br>(CTCCA) | GATGG<br>(CCATC) | GGCGG<br>(CCGCC) |
| AAACA<br>(TGTTT) | ATACC<br>(GGTAT) | AGTGA<br>(TCACT) | ACGTC<br>(GACGT) | TACCG<br>(CGGTA) | TGGAC<br>(GTCCA) | GATGC<br>(GCATC) | GGCGC<br>(GCGCC) |
| AAACT<br>(AGTTT) | ATTAA<br>(TTAAT) | AGTGT<br>(ACACT) | ACGGA<br>(TCCGT) | TACCC<br>(GGGTA) | TGGTG<br>(CACCA) | GATCG<br>(CGATC) | GGCCG<br>(CGGCC) |
| AAACG<br>(CGTTT) | ATTAG<br>(CTAAT) | AGTGG<br>(CCACT) | ACGGT<br>(ACCGT) | TTAAA<br>(TTTAA) | TGGTC<br>(GACCA) | GATCC<br>(GGATC) | GCAAG<br>(CTTGC) |
| AAACC<br>(GGTTT) | ATTAC<br>(GTAAT) | AGTGC<br>(GCACT) | ACGGG<br>(CCCGT) | TTAAG<br>(CTTAA) | TGGGA<br>(TCCCA) | GAGAG<br>(CTCTC) | GCATG<br>(CATGC) |
| AATAA<br>(TTATT) | ATTTA<br>(TAAAT) | AGTCA<br>(TGACT) | ACGGC<br>(GCCGT) | TTAAC<br>(GTTAA) | TGGGG<br>(CCCCA) | GAGAC<br>(GTCTC) | GCAGG<br>(CCTGC) |
| AATAT<br>(ATATT) | ATTTG<br>(CAAAT) | AGTCG<br>(CGACT) | ACGCA<br>(TGCGT) | TTATG<br>(CATAA) | TGGGC<br>(GCCCA) | GAGTG<br>(CACTC) | GCAGC<br>(GCTGC) |
| AATAG<br>(CTATT) | ATTTC<br>(GAAAT) | AGTCC<br>(GGACT) | ACGCG<br>(CGCGT) | TTATC<br>(GATAA) | TGGCA<br>(TGCCA) | GAGTC<br>(GACTC) | GCACG<br>(CGTGC) |
| AATAC | ATTGA | AGGAA | ACGCC | TTAGA | TGGCG | GAGGG | GCTAG |

| | | | | | | | |
|---|---|---|---|---|---|---|---|
| (GTATT) | (TCAAT) | (TTCCT) | (GGCGT) | (TCTAA) | (CGCCA) | (CCCTC) | (CTAGC) |
| AATTA<br>(TAATT) | ATTGT<br>(ACAAT) | AGGAG<br>(CTCCT) | ACCAA<br>(TTGGT) | TTAGG<br>(CCTAA) | TGGCC<br>(GGCCA) | GAGGC<br>(GCCTC) | GCTTG<br>(CAAGC) |
| AATTG<br>(CAATT) | ATTGG<br>(CCAAT) | AGGAC<br>(GTCCT) | ACCAG<br>(CTGGT) | TTAGC<br>(GCTAA) | TGCAG<br>(CTGCA) | GAGCG<br>(CGCTC) | GCTGG<br>(CCAGC) |
| AATTC<br>(GAATT) | ATTGC<br>(GCAAT) | AGGTA<br>(TACCT) | ACCAC<br>(GTGGT) | TTACA<br>(TGTAA) | TGCAC<br>(GTGCA) | GAGCC<br>(GGCTC) | GCTCG<br>(CGAGC) |
| AATGA<br>(TCATT) | ATTCA<br>(TGAAT) | AGGTG<br>(CACCT) | ACCTA<br>(TAGGT) | TTACG<br>(CGTAA) | TGCTG<br>(CAGCA) | GACAG<br>(CTGTC) | GCGAG<br>(CTCGC) |
| AATGT<br>(ACATT) | ATTCT<br>(AGAAT) | AGGTC<br>(GACCT) | ACCTG<br>(CAGGT) | TTACC<br>(GGTAA) | TGCTC<br>(GAGCA) | GACAC<br>(GTGTC) | GCGTG<br>(CACGC) |
| AATGG<br>(CCATT) | ATTCG<br>(CGAAT) | AGGGA<br>(TCCCT) | ACCTC<br>(GAGGT) | TTTAG<br>(CTAAA) | TGCGA<br>(TCGCA) | GACTG<br>(CAGTC) | GCGGG<br>(CCCGC) |
| AATGC<br>(GCATT) | ATTCC<br>(GGAAT) | AGGGT<br>(ACCCT) | ACCGA<br>(TCGGT) | TTTAC<br>(GTAAA) | TGCGG<br>(CCGCA) | GACGG<br>(CCGTC) | GCGGC<br>(GCCGC) |
| AATCA<br>(TGATT) | ATGAA<br>(TTCAT) | AGGGG<br>(CCCCT) | ACCGG<br>(CCGGT) | TTTTG<br>(CAAAA) | TGCGC<br>(GCGCA) | GACGC<br>(GCGTC) | GCGCG<br>(CGCGC) |
| AATCT<br>(AGATT) | ATGAT<br>(ATCAT) | AGGGC<br>(GCCCT) | ACCGC<br>(GCGGT) | TTTTC<br>(GAAAA) | TGCCG<br>(CGGCA) | GACCG<br>(CGGTC) | GCCAG<br>(CTGGC) |
| AATCG<br>(CGATT) | ATGAG<br>(CTCAT) | AGGCA<br>(TGCCT) | ACCCA<br>(TGGGT) | TTTGA<br>(TCAAA) | TGCCC<br>(GGGCA) | GACCC<br>(GGGTC) | GCCTG<br>(CAGGC) |
| AATCC<br>(GGATT) | ATGAC<br>(GTCAT) | AGGCT<br>(AGCCT) | ACCCG<br>(CGGGT) | TTTGG<br>(CCAAA) | TCAAG<br>(CTTGA) | GTAAG<br>(CTTAC) | GCCGG<br>(CCGGC) |
| AAGAA<br>(TTCTT) | ATGTA<br>(TACAT) | AGGCG<br>(CGCCT) | ACCCC<br>(GGGGT) | TTTGC<br>(GCAAA) | TCAAC<br>(GTTGA) | GTAAC<br>(GTTAC) | GCCCG<br>(CGGGC) |
| AAGAT<br>(ATCTT) | ATGTG<br>(CACAT) | AGGCC<br>(GGCCT) | TAAAA<br>(TTTTA) | TTTCA<br>(TGAAA) | TCATG<br>(CATGA) | GTATG<br>(CATAC) | CAAAG<br>(CTTTG) |
| AAGAG<br>(CTCTT) | ATGTC<br>(GACAT) | AGCAA<br>(TTGCT) | TAAAG<br>(CTTTA) | TTTCG<br>(CGAAA) | TCATC<br>(GATGA) | GTAGG<br>(CCTAC) | CAATG<br>(CATTG) |
| AAGAC<br>(GTCTT) | ATGGA<br>(TCCAT) | AGCAG<br>(CTGCT) | TAAAC<br>(GTTTA) | TTTCC<br>(GGAAA) | TCAGA<br>(TCTGA) | GTAGC<br>(GCTAC) | CAAGG<br>(CCTTG) |
| AAGTA<br>(TACTT) | ATGGT<br>(ACCAT) | AGCAC<br>(GTGCT) | TAATA<br>(TATTA) | TTGAA<br>(TTCAA) | TCAGG<br>(CCTGA) | GTACG<br>(CGTAC) | CAACG<br>(CGTTG) |
| AAGTT<br>(AACTT) | ATGGG<br>(CCCAT) | AGCTA<br>(TAGCT) | TAATG<br>(CATTA) | TTGAG<br>(CTCAA) | TCAGC<br>(GCTGA) | GTACC<br>(GGTAC) | CATAG<br>(CTATG) |
| AAGTG<br>(CACTT) | ATGGC<br>(GCCAT) | AGCTG<br>(CAGCT) | TAATC<br>(GATTA) | TTGAC<br>(GTCAA) | TCACG<br>(CGTGA) | GTTAG<br>(CTAAC) | CATGG<br>(CCATG) |
| AAGTC<br>(GACTT) | ATGCA<br>(TGCAT) | AGCTC<br>(GAGCT) | TAAGA<br>(TCTTA) | TTGTG<br>(CACAA) | TCACC<br>(GGTGA) | GTTTG<br>(CAAAC) | CATCG<br>(CGATG) |
| AAGGA<br>(TCCTT) | ATGCT<br>(AGCAT) | AGCGA<br>(TCGCT) | TAAGG<br>(CCTTA) | TTGTC<br>(GACAA) | TCTAG<br>(CTAGA) | GTTGG<br>(CCAAC) | CAGAG<br>(CTCTG) |
| AAGGT<br>(ACCTT) | ATGCG<br>(CGCAT) | AGCGT<br>(ACGCT) | TAAGC<br>(GCTTA) | TTGGA<br>(TCCAA) | TCTAC<br>(GTAGA) | GTTGC<br>(GCAAC) | CAGTG<br>(CACTG) |
| AAGGG<br>(CCCTT) | ATGCC<br>(GGCAT) | AGCGG<br>(CCGCT) | TAACA<br>(TGTTA) | TTGGG<br>(CCCAA) | TCTTG<br>(CAAGA) | GTTCG<br>(CGAAC) | CAGGG<br>(CCCTG) |
| AAGGC<br>(GCCTT) | ATCAA<br>(TTGAT) | AGCGC<br>(GCGCT) | TAACG<br>(CGTTA) | TTGGC<br>(GCCAA) | TCTTC<br>(GAAGA) | GTTCC<br>(GGAAC) | CAGCG<br>(CGCTG) |
| AAGCA<br>(TGCTT) | ATCAG<br>(CTGAT) | AGCCA<br>(TGGCT) | TAACC<br>(GGTTA) | TTGCA<br>(TGCAA) | TCTGG<br>(CCAGA) | GTGAG<br>(CTCAC) | CACAG<br>(CTGTG) |

| | | | | | | | |
|---|---|---|---|---|---|---|---|
| AAGCT (AGCTT) | ATCAC (GTGAT) | AGCCG (CGGCT) | TATAA (TTATA) | TTGCG (CGCAA) | TCTGC (GCAGA) | GTGAC (GTCAC) | CACGG (CCGTG) |
| AAGCG (CGCTT) | ATCTA (TAGAT) | AGCCC (GGGCT) | TATAG (CTATA) | TTGCC (GGCAA) | TCTCG (CGAGA) | GTGTG (CACAC) | CACCG (CGGTG) |
| AAGCC (GGCTT) | ATCTG (CAGAT) | ACAAA (TTTGT) | TATAC (GTATA) | TTCAG (CTGAA) | TCTCC (GGAGA) | GTGGG (CCCAC) | CTAAG (CTTAG) |
| AACAA (TTGTT) | ATCTC (GAGAT) | ACAAG (CTTGT) | TATTG (CAATA) | TTCAC (GTGAA) | TCGAG (CTCGA) | GTGGC (GCCAC) | CTAGG (CCTAG) |
| AACAT (ATGTT) | ATCGA (TCGAT) | ACAAC (GTTGT) | TATTC (GAATA) | TTCTG (CAGAA) | TCGAC (GTCGA) | GTGCG (CGCAC) | CTACG (CGTAG) |
| AACAG (CTGTT) | ATCGT (ACGAT) | ACATA (TATGT) | TATGA (TCATA) | TTCTC (GAGAA) | TCGTG (CACGA) | GTGCC (GGCAC) | CTTGG (CCAAG) |
| AACAC (GTGTT) | ATCGG (CCGAT) | ACATG (CATGT) | TATGG (CCATA) | TTCGA (TCGAA) | TCGTC (GACGA) | GTCAG (CTGAC) | CTTCG (CGAAG) |
| AACTA (TAGTT) | ATCGC (GCGAT) | ACATC (GATGT) | TATGC (GCATA) | TTCGG (CCGAA) | TCGGA (TCCGA) | GTCTG (CAGAC) | CTGAG (CTCAG) |
| AACTG (CAGTT) | ATCCA (TGGAT) | ACAGA (TCTGT) | TATCA (TGATA) | TTCGC (GCGAA) | TCGGG (CCCGA) | GTCGG (CCGAC) | CTGGG (CCCAG) |
| AACTC (GAGTT) | ATCCT (AGGAT) | ACAGT (ACTGT) | TATCG (CGATA) | TTCCA (TGGAA) | TCGGC (GCCGA) | GTCGC (GCGAC) | CTGCG (CGCAG) |
| AACGA (TCGTT) | ATCCG (CGGAT) | ACAGG (CCTGT) | TATCC (GGATA) | TTCCG (CGGAA) | TCGCG (CGCGA) | GTCCG (CGGAC) | CTCGG (CCGAG) |
| AACGT (ACGTT) | ATCCC (GGGAT) | ACAGC (GCTGT) | TAGAA (TTCTA) | TTCCC (GGGAA) | TCGCC (GGCGA) | GTCCC (GGGAC) | CTCCG (CGGAG) |
| AACGG (CCGTT) | AGAAA (TTTCT) | ACACA (TGTGT) | TAGAG (CTCTA) | TGAAG (CTTCA) | TCCAG (CTGGA) | GGAAG (CTTCC) | CGAGG (CCTCG) |
| AACGC (GCGTT) | AGAAG (CTTCT) | ACACG (CGTGT) | TAGAC (GTCTA) | TGAAC (GTTCA) | TCCAC (GTGGA) | GGATG (CATCC) | CGACG (CGTCG) |
| AACCA (TGGTT) | AGAAC (GTTCT) | ACACC (GGTGT) | TAGTA (TACTA) | TGATG (CATCA) | TCCTG (CAGGA) | GGAGG (CCTCC) | CGTGG (CCACG) |
| AACCT (AGGTT) | AGATA (TATCT) | ACTAA (TTAGT) | TAGTG (CACTA) | TGATC (GATCA) | TCCTC (GAGGA) | GGAGC (GCTCC) | CGGGG (CCCCG) |
| AACCG (CGGTT) | AGATG (CATCT) | ACTAG (CTAGT) | TAGTC (GACTA) | TGAGA (TCTCA) | TCCGG (CCGGA) | GGACG (CGTCC) | CGGCG (CGCCG) |
| AACCC (GGGTT) | AGATC (GATCT) | ACTAC (GTAGT) | TAGGA (TCCTA) | TGAGG (CCTCA) | TCCGC (GCGGA) | GGACC (GGTCC) | CGCGG (CCGCG) |
| ATAAA (TTTAT) | AGAGA (TCTCT) | ACTTA (TAAGT) | TAGGG (CCCTA) | TGAGC (GCTCA) | TCCCG (CGGGA) | GGTAG (CTACC) | CCAGG (CCTGG) |
| ATAAT (ATTAT) | AGAGT (ACTCT) | ACTTG (CAAGT) | TAGGC (GCCTA) | TGACA (TGTCA) | TCCCC (GGGGA) | GGTTG (CAACC) | CCGGG (CCCGG) |

**Supplementary Table S1**. Tabulation of all oligonucleotides of length *n* = 2,3,4 and 5. For *n* = 2 and 4, self-complementary oligonucleotides are listed in separate tables. Each of all other oligonucleotides is listed in pair with its reverse complementary one within the same cell. For any given *n*, the elements of the corresponding GGS vector are the *odds ratios* of the oligonucleotides listed outside brackets (for more details, see Methods).

# k-mer based genome analysis

For each chromosome in our collection we concatenate each sequence with its reverse complement and then compute the observed frequencies of all k-mers (namely, oligonucleotides of a given length), for k = 2, 3, 4, and 5. In the case of bacterial genomes with more than one chromosomes, we take into account only the longest one. For each value of k, the k-mer frequencies constitute the elements of a vector which we assign to the corresponding genome. These vectors include only one from each pair of non self-complementary k-mers and the self-complementary ones. As described in the main text, for k = 2, 3, 4 and 5, the numbers of elements of the corresponding vectors are w2 = 10, w3 = 32, w4 = 136 and w5 = 512.
In order to assess the efficacy of observed k-mer frequencies in distinguishing between genomes of different evolutionary descent, we perform classification analysis of bacterial genomes based on their corresponding k-mer vectors. As in the case of GGSs-based analysis, we use the J48, SMO and LMT classifiers in order to predict in which phylum or class the bacterial species represented in our collection belong. These experiments correspond to a coarse classification of bacteria according to their major taxonomic subdivisions. We present our results in **Supplementary Table S2**.

| | TP Rate | FP Rate | Precision | Recall | F-Measure | MCC | ROC Area |
|---|---|---|---|---|---|---|---|
| **J48** | | | | | | | |
| k=2 | 0,800 | 0,030 | 0,794 | 0,799 | 0,795 | 0,768 | 0,903 |
| k=3 | 0,822 | 0,023 | 0,822 | 0,822 | 0,821 | 0,799 | 0,911 |
| k=4 | 0,851 | 0,019 | 0,851 | 0,851 | 0,851 | 0,832 | 0,925 |
| k=5 | 0,848 | 0,019 | 0,852 | 0,848 | 0,849 | 0,831 | 0,923 |
| **SMO** | | | | | | | |
| k=2 | 0,662 | 0,087 | 0,665 | 0,662 | 0,636 | 0,597 | 0,873 |
| k=3 | 0,810 | 0,041 | 0,813 | 0,810 | 0,799 | 0,779 | 0,951 |
| k=4 | 0,939 | 0,011 | 0,940 | 0,939 | 0,938 | 0,930 | 0,986 |
| k=5 | 0,975 | 0,004 | 0,976 | 0,975 | 0,975 | 0,972 | 0,994 |
| **LMT** | | | | | | | |
| k=2 | 0,824 | 0,026 | 0,822 | 0,824 | 0,823 | 0,799 | 0,941 |
| k=3 | 0,907 | 0,014 | 0,907 | 0,907 | 0,906 | 0,894 | 0,979 |
| k=4 | 0,965 | 0,005 | 0,966 | 0,965 | 0,965 | 0,961 | 0,996 |
| k=5 | 0,970 | 0,004 | 0,971 | 0,970 | 0,971 | 0,967 | 0,996 |

**Supplementary Table S2. Classification of bacteria based on the vectors of observed k-mer frequencies.** Weighted average statistics denoting the

performance of the classifiers (J48, SMO, LMT) we applied to our dataset of observed k-mer frequencies, for k = 2, 3, 4 and 5. **For further explanations on the statistics, see legend of Table 1.**

To further evaluate whether the observed k-mer frequencies correspond to phylogenetic traits which are distinct among bacteria species, we calculate for each value of k the Manhattan distance between all pairs of the corresponding k-mer vectors. Based on these distances, we construct the cladograms of the species belonging to the same phylum or class via complete-linkage hierarchical clustering. Then, using the web-based tool Compare2Trees, we compare the k-mer based cladograms with the species trees we retrieved from NCBI Taxonomy. **Supplementary Figure S1** depicts the resulting scores, which express the percent topological similarity of the trees in comparison.
For discussion on these results and a comparative evaluation with respect to the corresponding GGSs-based analysis, see sections 'Reconstruction of Bacterial Phylogeny' and 'Concluding remarks and perspectives'.

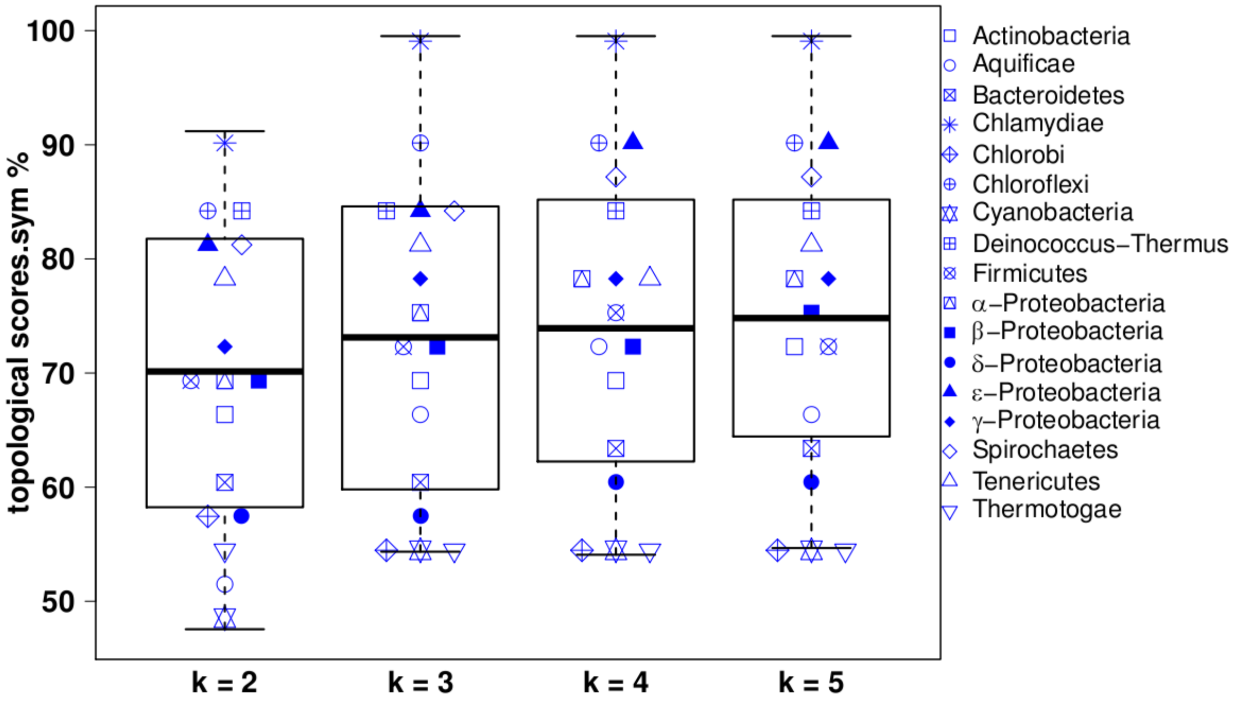


**Supplementary Figure S1.** Boxplots of topological scores. For each phylum or class, the scores reflect the percent topological similarity of the k-mer based trees and species trees. The higher the scores, the more accurately k-mer vectors capture the phylogenetic relationships between bacteria. Observed k-mer frequencies are calculated for k = 2, 3, 4 and 5.

# CNE, exon and surrogate datasets

| DATASET NAME | DATASET LENGTH [in nucleotides] | Number of concatenated elements |
|---|---|---|
| CNE 75-80 | 2,509,079 | 10,133 |
| CNE 80-85 | 1,911,523 | 7,617 |
| CNE 90-95 | 1,493,461 | 5,860 |
| CNE 95-100 (UCNEs) | 1,428,961 | 4,386 |
| Amniotic CNEs | 4,864,765 | 16,575 |
| Mammalian CNEs 1 | 10,152,359 | 41,167 |
| Mammalian CNEs 2 | 10,096,043 | 41,167 |
| Exon collection 1 | 10,631,246 | 64,000 |
| Exon collection 2 | 10,802,082 | 64,000 |
| Exon collection 3 | 10,843,406 | 63,786 |

For each one of the above datasets a 'surrogate' one is composed, by concatenating an equal number of segments taken from the non-constrained repeat-masked part of the human genome. The lengths of these segments are equal to the lengths of their counterparts in the original dataset.

# Naïve visualisation of the tendency of sequences to form clusters in the space spanned by GGSs according to their functionality and degree of conservation.

A first naïve search on whether the GGS vectors cluster according to the specific function of the corresponding datasets (which are concatenates of sequence stretches of the same type) is attempted in the following way. For every value of n (n = 2, 3, 4, 5) the Manhattan distances of every two of the twenty-two vectors corresponding to the datasets composed and used herein are computed (see in the Methods). Then, the obtained 231 (22x21/2) values of distance are sorted in decreasing order for each value of n and tabulated in the Supplementary Information. The character D (stands for 'different') or S (stands for for 'similar') is put as the first character in each line of the composed tables. Lines correspond to the computed distance values for each pair of datasets. Pairs of datasets characterized as 'similar' (S) are: both exon (e*e), both CNE (c*c) or both surrogate sequences (r*r). We remind that the elements composing the later sequence concatenates (datasets denoted by r) are picked at random from the non-constrained, non-repeated part of the genome. All other cases of pairwise distances (e*c or c*e, e*r or r*e, c*r or r*c) are characterized 'different' (D). We observe that for most values of n, almost all pairs of *similar* datasets have lower mutual distances than any pairs of *different* datasets. This holds true for n=3, 4, 5 where the deviations from this rule are one, three and four respectively. All these deviating cases are distances in pairs of CNEs (c*c) which include one dataset of 'mammalian CNEs'. As we commented in the Methods section, 'mammalian' are the CNEs selected under the less strict conditions of sequence conservation. Thus, these pairs of datasets, although both CNEs, are expected to exhibit relatively high mutual distances, as one of the two datasets abides less than the other to the conjectured compositional similarity of constrained CNE sequences. In the case of genomic signatures of first neighbours (n=2), the deviations are slightly more (13 cases). Here again, almost all (all except one) are of the type described above, i.e. c*c including one mammalian dataset.
This naïve estimation of the tendency of GGS vectors of functionally distinct datasets to cluster according to their functionalities has the advantage of being independent of any elaborate feature of the existing clustering algorithms. It points out in a convincing way that such a clustering does exist. Moreover, we point out that this clustering ability is higher for n-words longer than the dinucleotides, considered so far as the more relevant units in the composition of genomes.

# DISTANCES FOR ALL PAIRS OF DATASETS FOR DUPLETS

THERE ARE ONLY 13/231 DEVIATIONS FROM THE PERFECT FRACTIONATION BETWEEN COMPARISONS OF 'DIFFERENT (D)' (c*e OR e*c, r*c OR c*r, e*r OR r*e) (UPPER LAYER, LONG Delta-DISTANCE) AND OF 'SIMILAR (S)' (c*c, e*e, r*r) (LOWER LAYER, SHORT Delta-DISTANCE) DATASETS. c, e, r STAND FOR CNEs, EXONS AND RANDOMLY CHOSEN SEGMENTS (SURROGATES). 12 OUT OF THE 13 DEVIATIONS INVOLVE 'mammalian_CNEs'.

| D-OR-S | DATASETS WHOSE Delta-DISTANCE IS COMPUTED | | | Delta-DISTANCE |
|---|---|---|---|---|
| D\|e*c | h_exons_2 | - | ucnes95-100 | 1.105600 |
| D\|e*c | h_exons_1 | - | ucnes95-100 | 1.052800 |
| D\|c*e | cnes90-95 | - | h_exons_2 | 1.041700 |
| D\|e*c | h_exons_2 | - | mammalian_CNEs_2 | 1.026300 |
| D\|c*e | amniotic CNEs | - | h_exons_2 | 1.017700 |
| D\|e*c | h_exons_3 | - | ucnes95-100 | 1.011600 |
| D\|e*c | h_exons_2 | - | mammalian_CNEs_1 | 1.000000 |
| D\|c*e | cnes85-90 | - | h_exons_2 | 0.991400 |
| D\|c*e | cnes90-95 | - | h_exons_1 | 0.991100 |
| D\|c*e | cnes80-85 | - | h_exons_2 | 0.950300 |
| D\|c*e | cnes90-95 | - | h_exons_3 | 0.949500 |
| D\|e*r | h_exons_2 | - | ucnes95-100_rnd | 0.943700 |
| D\|c*e | amniotic CNEs | - | h_exons_1 | 0.938500 |
| D\|e*c | h_exons_1 | - | mammalian_CNEs_2 | 0.935700 |
| D\|e*r | h_exons_2 | - | mammalian_rnd_2 | 0.933900 |
| D\|r*e | cnes75-80_rnd | - | h_exons_2 | 0.933400 |
| D\|e*r | h_exons_2 | - | h_exons_rnd_2 | 0.930100 |
| D\|e*r | h_exons_2 | - | h_exons_rnd_3 | 0.928900 |
| D\|r*e | amniotic_rnd | - | h_exons_2 | 0.928300 |
| D\|r*e | cnes90-95_rnd | - | h_exons_2 | 0.925700 |
| D\|e*r | h_exons_2 | - | h_exons_rnd_1 | 0.925500 |
| D\|e*r | h_exons_2 | - | mammalian_rnd_1 | 0.925400 |
| D\|c*e | cnes85-90 | - | h_exons_1 | 0.919800 |
| D\|r*e | cnes80-85_rnd | - | h_exons_2 | 0.917900 |
| D\|r*e | cnes85-90_rnd | - | h_exons_2 | 0.917300 |
| D\|e*c | h_exons_1 | - | mammalian_CNEs_1 | 0.909400 |
| D\|c*e | cnes75-80 | - | h_exons_2 | 0.909300 |
| D\|e*c | h_exons_3 | - | mammalian_CNEs_2 | 0.902700 |
| D\|c*e | amniotic CNEs | - | h_exons_3 | 0.893100 |
| D\|c*e | cnes85-90 | - | h_exons_3 | 0.883200 |
| D\|e*c | h_exons_3 | - | mammalian_CNEs_1 | 0.876400 |
| D\|c*e | cnes80-85 | - | h_exons_1 | 0.869900 |
| D\|e*r | h_exons_1 | - | ucnes95-100_rnd | 0.864500 |
| D\|e*r | h_exons_1 | - | mammalian_rnd_2 | 0.854700 |
| D\|r*e | cnes75-80_rnd | - | h_exons_1 | 0.854200 |
| D\|e*r | h_exons_1 | - | h_exons_rnd_2 | 0.850900 |
| D\|e*r | h_exons_1 | - | h_exons_rnd_3 | 0.849700 |

```
D|r*e |   amniotic_rnd       -  h_exons_1          |       0.849100
D|r*e |   cnes90-95_rnd      -  h_exons_1          |       0.846500
D|e*r |   h_exons_1          -  h_exons_rnd_1      |       0.846300
D|e*r |   h_exons_1          -  mammalian_rnd_1    |       0.846200
D|c*e |   cnes80-85          -  h_exons_3          |       0.842100
D|r*e |   cnes80-85_rnd      -  h_exons_1          |       0.838700
D|r*e |   cnes85-90_rnd      -  h_exons_1          |       0.838100
D|c*e |   cnes75-80          -  h_exons_1          |       0.830100
D|e*r |   h_exons_3          -  ucnes95-100_rnd    |       0.819100
D|e*r |   h_exons_3          -  mammalian_rnd_2    |       0.809300
D|r*e |   cnes75-80_rnd      -  h_exons_3          |       0.808800
D|e*r |   h_exons_3          -  h_exons_rnd_2      |       0.805500
D|e*r |   h_exons_3          -  h_exons_rnd_3      |       0.804300
D|r*e |   amniotic_rnd       -  h_exons_3          |       0.803700
D|r*e |   cnes90-95_rnd      -  h_exons_3          |       0.801100
D|e*r |   h_exons_3          -  h_exons_rnd_1      |       0.800900
D|e*r |   h_exons_3          -  mammalian_rnd_1    |       0.800800
D|c*e |   cnes75-80          -  h_exons_3          |       0.796100
D|r*e |   cnes80-85_rnd      -  h_exons_3          |       0.793300
D|r*e |   cnes85-90_rnd      -  h_exons_3          |       0.792700
D|r*c |   cnes90-95_rnd      -  ucnes95-100        |       0.732900
D|r*c |   amniotic_rnd       -  ucnes95-100        |       0.725300
D|r*c |   cnes85-90_rnd      -  ucnes95-100        |       0.725100
D|r*c |   mammalian_rnd_2    -  ucnes95-100        |       0.720300
D|r*c |   cnes75-80_rnd      -  ucnes95-100        |       0.720000
D|c*r |   ucnes95-100        -  ucnes95-100_rnd    |       0.718300
D|r*c |   h_exons_rnd_2      -  ucnes95-100        |       0.715700
D|r*c |   mammalian_rnd_1    -  ucnes95-100        |       0.714200
D|r*c |   h_exons_rnd_1      -  ucnes95-100        |       0.713900
S|c*c |   mammalian_CNEs_2   -  ucnes95-100        |       0.704900 #01
D|r*c |   cnes80-85_rnd      -  ucnes95-100        |       0.702300
D|r*c |   h_exons_rnd_3      -  ucnes95-100        |       0.701900
D|c*r |   cnes90-95          -  cnes90-95_rnd      |       0.647000
D|r*c |   amniotic_rnd       -  cnes90-95          |       0.642200
D|r*c |   cnes85-90_rnd      -  cnes90-95          |       0.641800
D|r*c |   cnes75-80_rnd      -  cnes90-95          |       0.635900
D|c*r |   cnes90-95          -  mammalian_rnd_2    |       0.633600
D|c*r |   cnes90-95          -  ucnes95-100_rnd    |       0.630200
D|c*r |   cnes90-95          -  mammalian_rnd_1    |       0.629900
S|c*c |   mammalian_CNEs_1   -  ucnes95-100        |       0.628400 #02
D|c*r |   cnes90-95          -  h_exons_rnd_2      |       0.627600
D|c*r |   cnes90-95          -  h_exons_rnd_1      |       0.626800
D|r*c |   cnes80-85_rnd      -  cnes90-95          |       0.621400
D|c*r |   cnes90-95          -  h_exons_rnd_3      |       0.616000
S|c*c |   cnes90-95          -  mammalian_CNEs_2   |       0.602400 #03
D|c*r |   cnes85-90          -  cnes90-95_rnd      |       0.597900
D|r*c |   amniotic_rnd       -  cnes85-90          |       0.593700
D|c*r |   cnes85-90          -  cnes85-90_rnd      |       0.590700
```

```
D|r*c |   cnes75-80_rnd      -  cnes85-90          |       0.588000
D|c*r |   cnes85-90          -  mammalian_rnd_2    |       0.584300
D|c*r |   cnes85-90          -  mammalian_rnd_1    |       0.578200
D|c*r |   cnes85-90          -  ucnes95-100_rnd    |       0.577900
D|c*r |   cnes85-90          -  h_exons_rnd_1      |       0.575100
D|c*r |   cnes85-90          -  h_exons_rnd_2      |       0.573300
D|r*c |   cnes80-85_rnd      -  cnes85-90          |       0.573100
D|c*r |   cnes85-90          -  h_exons_rnd_3      |       0.568100
D|c*r |   cnes80-85          -  cnes90-95_rnd      |       0.559000
D|r*c |   amniotic_rnd       -  cnes80-85          |       0.554800
D|c*r |   cnes80-85          -  cnes85-90_rnd      |       0.551800
D|r*c |   cnes75-80_rnd      -  cnes80-85          |       0.549100
D|c*r |   cnes80-85          -  mammalian_rnd_2    |       0.545400
S|c*c |   cnes90-95          -  mammalian_CNEs_1   |       0.540300 #04
D|c*r |   cnes80-85          -  mammalian_rnd_1    |       0.539300
D|c*r |   cnes80-85          -  ucnes95-100_rnd    |       0.539000
D|c*r |   cnes80-85          -  h_exons_rnd_1      |       0.536200
D|c*r |   cnes80-85          -  h_exons_rnd_2      |       0.534400
D|c*r |   cnes80-85          -  cnes80-85_rnd      |       0.534200
D|c*r |   cnes80-85          -  h_exons_rnd_3      |       0.529200
S|c*c |   cnes85-90          -  mammalian_CNEs_2   |       0.520100 #05
D|c*r |   amniotic CNEs      -  amniotic_rnd       |       0.512400
D|c*r |   cnes75-80          -  cnes90-95_rnd      |       0.512000
D|c*r |   amniotic CNEs      -  cnes90-95_rnd      |       0.509400
D|r*c |   amniotic_rnd       -  cnes75-80          |       0.507800
D|c*r |   cnes75-80          -  cnes85-90_rnd      |       0.504800
D|c*r |   amniotic CNEs      -  cnes75-80_rnd      |       0.504700
D|c*r |   amniotic CNEs      -  cnes85-90_rnd      |       0.502800
D|c*r |   cnes75-80          -  cnes75-80_rnd      |       0.502100
D|c*r |   amniotic CNEs      -  mammalian_rnd_2    |       0.499800
D|c*r |   cnes75-80          -  mammalian_rnd_2    |       0.498400
D|c*r |   amniotic CNEs      -  ucnes95-100_rnd    |       0.496200
D|c*r |   amniotic CNEs      -  mammalian_rnd_1    |       0.494100
D|c*r |   cnes75-80          -  mammalian_rnd_1    |       0.492300
D|c*r |   cnes75-80          -  ucnes95-100_rnd    |       0.492000
D|c*r |   cnes75-80          -  h_exons_rnd_1      |       0.489200
D|c*r |   amniotic CNEs      -  cnes80-85_rnd      |       0.488800
D|c*r |   cnes75-80          -  h_exons_rnd_2      |       0.487400
D|c*r |   cnes75-80          -  cnes80-85_rnd      |       0.487200
D|c*r |   amniotic CNEs      -  h_exons_rnd_1      |       0.487000
D|c*r |   amniotic CNEs      -  h_exons_rnd_2      |       0.486000
D|c*r |   cnes75-80          -  h_exons_rnd_3      |       0.482200
D|c*r |   amniotic CNEs      -  h_exons_rnd_3      |       0.481400
S|c*c |   cnes85-90          -  mammalian_CNEs_1   |       0.477400 #06
S|c*c |   cnes80-85          -  mammalian_CNEs_2   |       0.470800 #07
S|c*c |   amniotic CNEs      -  mammalian_CNEs_2   |       0.467800 #08
S|c*c |   cnes80-85          -  mammalian_CNEs_1   |       0.423900 #09
S|c*c |   cnes75-80          -  mammalian_CNEs_2   |       0.400800 #10
```

```
D|r*c |   amniotic_rnd       -  mammalian_CNEs_2  |       0.386600
D|r*c |   cnes75-80_rnd      -  mammalian_CNEs_2  |       0.379700
D|r*c |   cnes85-90_rnd      -  mammalian_CNEs_2  |       0.374800
D|c*r |   mammalian_CNEs_2   -  mammalian_rnd_2   |       0.373200
S|c*c |   amniotic CNEs      -  mammalian_CNEs_1  |       0.372700 #11
D|c*r |   mammalian_CNEs_2   -  ucnes95-100_rnd   |       0.372600
D|c*r |   mammalian_CNEs_2   -  mammalian_rnd_1   |       0.372500
D|r*c |   cnes90-95_rnd      -  mammalian_CNEs_2  |       0.372400
D|r*c |   cnes80-85_rnd      -  mammalian_CNEs_2  |       0.367400
D|r*c |   h_exons_rnd_1      -  mammalian_CNEs_2  |       0.360800
S|c*c |   cnes75-80          -  mammalian_CNEs_1  |       0.360300 #12
D|r*c |   h_exons_rnd_2      -  mammalian_CNEs_2  |       0.355200
D|r*c |   h_exons_rnd_3      -  mammalian_CNEs_2  |       0.354400
D|r*c |   amniotic_rnd       -  mammalian_CNEs_1  |       0.322100
D|r*c |   cnes90-95_rnd      -  mammalian_CNEs_1  |       0.315100
D|r*c |   cnes75-80_rnd      -  mammalian_CNEs_1  |       0.314400
D|r*c |   cnes85-90_rnd      -  mammalian_CNEs_1  |       0.312500
D|c*r |   mammalian_CNEs_1   -  mammalian_rnd_2   |       0.309500
S|c*c |   cnes75-80          -  ucnes95-100       |       0.306100 #13
D|c*r |   mammalian_CNEs_1   -  ucnes95-100_rnd   |       0.305900
D|r*c |   h_exons_rnd_2      -  mammalian_CNEs_1  |       0.304500
D|c*r |   mammalian_CNEs_1   -  mammalian_rnd_1   |       0.303800
D|r*c |   h_exons_rnd_1      -  mammalian_CNEs_1  |       0.299300
D|r*c |   h_exons_rnd_3      -  mammalian_CNEs_1  |       0.299300
D|r*c |   cnes80-85_rnd      -  mammalian_CNEs_1  |       0.298500
===============================================================
S|c*c |   amniotic CNEs      -  ucnes95-100       |       0.255700
S|c*c |   cnes80-85          -  ucnes95-100       |       0.254100
S|c*c |   cnes75-80          -  cnes90-95         |       0.208400
S|c*c |   cnes85-90          -  ucnes95-100       |       0.207200
S|c*c |   mammalian_CNEs_1   -  mammalian_CNEs_2  |       0.168300
S|c*c |   amniotic CNEs      -  cnes90-95         |       0.167600
S|c*c |   cnes90-95          -  ucnes95-100       |       0.139900
S|e*e |   h_exons_2          -  h_exons_3         |       0.137800
S|c*c |   amniotic CNEs      -  cnes80-85         |       0.133800
S|c*c |   cnes80-85          -  cnes90-95         |       0.131600
S|c*c |   cnes75-80          -  cnes85-90         |       0.129300
S|c*c |   amniotic CNEs      -  cnes75-80         |       0.128200
S|e*e |   h_exons_1          -  h_exons_2         |       0.119400
S|c*c |   amniotic CNEs      -  cnes85-90         |       0.118900
S|e*e |   h_exons_1          -  h_exons_3         |       0.096800
S|c*c |   cnes85-90          -  cnes90-95         |       0.082300
S|c*c |   cnes75-80          -  cnes80-85         |       0.077400
S|c*c |   cnes80-85          -  cnes85-90         |       0.066500
S|r*r |   cnes85-90_rnd      -  ucnes95-100_rnd   |       0.038600
S|r*r |   amniotic_rnd       -  h_exons_rnd_3     |       0.035000
S|r*r |   h_exons_rnd_3      -  ucnes95-100_rnd   |       0.034800
S|r*r |   amniotic_rnd       -  h_exons_rnd_2     |       0.034400
```

| | | | | | |
|---|---|---|---|---|---|
| S\|r*r | cnes90-95_rnd | - | ucnes95-100_rnd | \| | 0.034400 |
| S\|r*r | h_exons_rnd_2 | - | ucnes95-100_rnd | \| | 0.031800 |
| S\|r*r | cnes80-85_rnd | - | ucnes95-100_rnd | \| | 0.031600 |
| S\|r*r | cnes90-95_rnd | - | h_exons_rnd_3 | \| | 0.031600 |
| S\|r*r | cnes80-85_rnd | - | h_exons_rnd_2 | \| | 0.030800 |
| S\|r*r | cnes80-85_rnd | - | cnes90-95_rnd | \| | 0.030600 |
| S\|r*r | h_exons_rnd_1 | - | ucnes95-100_rnd | \| | 0.029000 |
| S\|r*r | cnes85-90_rnd | - | h_exons_rnd_2 | \| | 0.028600 |
| S\|r*r | amniotic_rnd | - | h_exons_rnd_1 | \| | 0.028200 |
| S\|r*r | amniotic_rnd | - | cnes80-85_rnd | \| | 0.027800 |
| S\|r*r | cnes85-90_rnd | - | h_exons_rnd_3 | \| | 0.027400 |
| S\|r*r | cnes90-95_rnd | - | h_exons_rnd_2 | \| | 0.026800 |
| S\|r*r | cnes75-80_rnd | - | cnes80-85_rnd | \| | 0.026300 |
| S\|r*r | h_exons_rnd_3 | - | mammalian_rnd_1 | \| | 0.025700 |
| S\|r*r | cnes75-80_rnd | - | h_exons_rnd_3 | \| | 0.025300 |
| S\|r*r | cnes85-90_rnd | - | mammalian_rnd_2 | \| | 0.025200 |
| S\|r*r | cnes75-80_rnd | - | h_exons_rnd_2 | \| | 0.024900 |
| S\|r*r | h_exons_rnd_2 | - | mammalian_rnd_1 | \| | 0.024900 |
| S\|r*r | cnes90-95_rnd | - | h_exons_rnd_1 | \| | 0.024200 |
| S\|r*r | amniotic_rnd | - | ucnes95-100_rnd | \| | 0.024000 |
| S\|r*r | cnes85-90_rnd | - | cnes90-95_rnd | \| | 0.024000 |
| S\|r*r | h_exons_rnd_3 | - | mammalian_rnd_2 | \| | 0.023800 |
| S\|r*r | amniotic_rnd | - | cnes90-95_rnd | \| | 0.023600 |
| S\|r*r | cnes90-95_rnd | - | mammalian_rnd_1 | \| | 0.023300 |
| S\|r*r | cnes80-85_rnd | - | h_exons_rnd_3 | \| | 0.023200 |
| S\|r*r | cnes80-85_rnd | - | mammalian_rnd_2 | \| | 0.023200 |
| S\|r*r | h_exons_rnd_2 | - | mammalian_rnd_2 | \| | 0.023200 |
| S\|r*r | cnes75-80_rnd | - | cnes85-90_rnd | \| | 0.023100 |
| S\|r*r | cnes80-85_rnd | - | cnes85-90_rnd | \| | 0.022800 |
| S\|r*r | cnes80-85_rnd | - | h_exons_rnd_1 | \| | 0.022200 |
| S\|r*r | cnes75-80_rnd | - | cnes90-95_rnd | \| | 0.021700 |
| S\|r*r | cnes75-80_rnd | - | ucnes95-100_rnd | \| | 0.021700 |
| S\|r*r | mammalian_rnd_1 | - | ucnes95-100_rnd | \| | 0.021500 |
| S\|r*r | amniotic_rnd | - | cnes85-90_rnd | \| | 0.020600 |
| S\|r*r | cnes85-90_rnd | - | h_exons_rnd_1 | \| | 0.020200 |
| S\|r*r | cnes85-90_rnd | - | mammalian_rnd_1 | \| | 0.019700 |
| S\|r*r | cnes75-80_rnd | - | h_exons_rnd_1 | \| | 0.019500 |
| S\|r*r | cnes80-85_rnd | - | mammalian_rnd_1 | \| | 0.018500 |
| S\|r*r | amniotic_rnd | - | mammalian_rnd_1 | \| | 0.018300 |
| S\|r*r | h_exons_rnd_1 | - | mammalian_rnd_2 | \| | 0.017600 |
| S\|r*r | cnes90-95_rnd | - | mammalian_rnd_2 | \| | 0.017200 |
| S\|r*r | mammalian_rnd_2 | - | ucnes95-100_rnd | \| | 0.017200 |
| S\|r*r | h_exons_rnd_1 | - | mammalian_rnd_1 | \| | 0.015700 |
| S\|r*r | h_exons_rnd_2 | - | h_exons_rnd_3 | \| | 0.014400 |
| S\|r*r | amniotic_rnd | - | mammalian_rnd_2 | \| | 0.014000 |
| S\|r*r | cnes75-80_rnd | - | mammalian_rnd_1 | \| | 0.013200 |
| S\|r*r | h_exons_rnd_1 | - | h_exons_rnd_3 | \| | 0.012800 |
| S\|r*r | mammalian_rnd_1 | - | mammalian_rnd_2 | \| | 0.012300 |

```
S|r*r |   amniotic_rnd      -  cnes75-80_rnd     |      0.011700
S|r*r |   cnes75-80_rnd     -  mammalian_rnd_2   |      0.010900
S|r*r |   h_exons_rnd_1     -  h_exons_rnd_2     |      0.010800

#####################################################################
```

## DISTANCES FOR ALL PAIRS OF DATASETS FOR TRIPLETS

THERE IS ONLY 1/231 DEVIATION FROM THE PERFECT FRACTIONATION BETWEEN COMPARISONS OF 'DIFFERENT (D)' (c*e OR e*c, r*c OR c*r, e*r OR r*e) (UPPER LAYER, LONG Delta-DISTANCE) AND OF 'SIMILAR (S)' (c*c, e*e, r*r) (LOWER LAYER, SHORT Delta-DISTANCE) DATASETS. c, e, r STAND FOR CNEs, EXONS AND RANDOMLY CHOSEN SEGMENTS (SURROGATES). THE UNIQUE DEVIATION INVOLVES 'mammalian_CNEs'.

```
D-OR-S|DATASETS WHOSE Delta-DISTANCE IS COMPUTED |Delta-DISTANCE
----------------------------------------------------------------
D|e*c |   h_exons_2         -  mammalian_CNEs_2  |      2.883500
D|e*c |   h_exons_1         -  mammalian_CNEs_2  |      2.793200
D|e*c |   h_exons_2         -  ucnes95-100       |      2.665800
D|e*c |   h_exons_3         -  mammalian_CNEs_2  |      2.643600
D|e*c |   h_exons_1         -  ucnes95-100       |      2.570900
D|e*c |   h_exons_2         -  mammalian_CNEs_1  |      2.561500
D|e*c |   h_exons_3         -  ucnes95-100       |      2.545300
D|c*e |   amniotic CNEs     -  h_exons_2         |      2.527000
D|c*e |   cnes90-95         -  h_exons_2         |      2.481600
D|e*c |   h_exons_1         -  mammalian_CNEs_1  |      2.453800
D|c*e |   amniotic CNEs     -  h_exons_3         |      2.426700
D|c*e |   amniotic CNEs     -  h_exons_1         |      2.423501
D|c*e |   cnes90-95         -  h_exons_1         |      2.345501
D|e*c |   h_exons_3         -  mammalian_CNEs_1  |      2.326200
D|c*e |   cnes90-95         -  h_exons_3         |      2.313300
D|r*c |   cnes85-90_rnd     -  ucnes95-100       |      2.260900
D|c*e |   cnes85-90         -  h_exons_2         |      2.260600
D|r*c |   mammalian_rnd_1   -  ucnes95-100       |      2.234600
D|c*r |   ucnes95-100       -  ucnes95-100_rnd   |      2.232500
D|r*c |   cnes75-80_rnd     -  ucnes95-100       |      2.220400
D|r*c |   amniotic_rnd      -  ucnes95-100       |      2.217600
D|r*c |   cnes80-85_rnd     -  ucnes95-100       |      2.217400
D|r*c |   h_exons_rnd_2     -  ucnes95-100       |      2.213900
D|r*c |   mammalian_rnd_2   -  ucnes95-100       |      2.208000
D|r*c |   h_exons_rnd_3     -  ucnes95-100       |      2.191400
D|r*c |   h_exons_rnd_1     -  ucnes95-100       |      2.164100
D|r*c |   cnes90-95_rnd     -  ucnes95-100       |      2.156500
D|r*e |   cnes85-90_rnd     -  h_exons_2         |      2.148900
D|c*e |   cnes85-90         -  h_exons_1         |      2.139300
D|c*e |   cnes85-90         -  h_exons_3         |      2.133700
```

| | | | | |
|---|---|---|---|---|
| D\|e*r | h_exons_2 | - | ucnes95-100_rnd | 2.129500 |
| D\|r*e | cnes75-80_rnd | - | h_exons_2 | 2.125400 |
| D\|e*r | h_exons_2 | - | mammalian_rnd_1 | 2.117800 |
| D\|r*e | cnes85-90_rnd | - | h_exons_1 | 2.107200 |
| D\|r*c | cnes85-90_rnd | - | cnes90-95 | 2.104300 |
| D\|e*r | h_exons_2 | - | mammalian_rnd_2 | 2.099600 |
| D\|e*r | h_exons_2 | - | h_exons_rnd_2 | 2.099500 |
| D\|r*e | cnes75-80_rnd | - | h_exons_1 | 2.090300 |
| D\|e*r | h_exons_2 | - | h_exons_rnd_3 | 2.089200 |
| D\|e*r | h_exons_1 | - | mammalian_rnd_1 | 2.088300 |
| D\|r*e | amniotic_rnd | - | h_exons_2 | 2.085400 |
| D\|r*e | cnes80-85_rnd | - | h_exons_2 | 2.085199 |
| D\|c*r | cnes90-95 | - | ucnes95-100_rnd | 2.084500 |
| D\|e*r | h_exons_1 | - | ucnes95-100_rnd | 2.081200 |
| D\|e*r | h_exons_2 | - | h_exons_rnd_1 | 2.070700 |
| D\|c*r | cnes90-95 | - | mammalian_rnd_1 | 2.070600 |
| D\|r*c | amniotic_rnd | - | cnes90-95 | 2.063400 |
| D\|e*r | h_exons_1 | - | h_exons_rnd_2 | 2.061400 |
| D\|e*r | h_exons_1 | - | mammalian_rnd_2 | 2.058500 |
| D\|r*c | cnes75-80_rnd | - | cnes90-95 | 2.058001 |
| D\|r*c | cnes80-85_rnd | - | cnes90-95 | 2.054000 |
| D\|e*r | h_exons_1 | - | h_exons_rnd_3 | 2.051900 |
| D\|c*r | cnes90-95 | - | h_exons_rnd_2 | 2.049900 |
| D\|c*r | cnes90-95 | - | mammalian_rnd_2 | 2.049000 |
| D\|c*e | cnes80-85 | - | h_exons_2 | 2.048600 |
| D\|r*e | amniotic_rnd | - | h_exons_1 | 2.044100 |
| D\|r*e | cnes80-85_rnd | - | h_exons_1 | 2.037899 |
| D\|e*r | h_exons_1 | - | h_exons_rnd_1 | 2.037400 |
| D\|c*r | cnes90-95 | - | h_exons_rnd_3 | 2.027800 |
| D\|r*e | cnes85-90_rnd | - | h_exons_3 | 2.016000 |
| D\|r*e | cnes90-95_rnd | - | h_exons_2 | 2.006900 |
| D\|c*r | cnes90-95 | - | cnes90-95_rnd | 2.006700 |
| D\|c*r | cnes90-95 | - | h_exons_rnd_1 | 1.996900 |
| D\|e*r | h_exons_3 | - | mammalian_rnd_1 | 1.991500 |
| D\|e*r | h_exons_3 | - | ucnes95-100_rnd | 1.990400 |
| D\|r*e | cnes75-80_rnd | - | h_exons_3 | 1.990100 |
| D\|c*e | cnes75-80 | - | h_exons_2 | 1.984400 |
| D\|c*r | amniotic CNEs | - | cnes85-90_rnd | 1.976700 |
| D\|r*e | cnes90-95_rnd | - | h_exons_1 | 1.973800 |
| D\|e*r | h_exons_3 | - | h_exons_rnd_2 | 1.965600 |
| D\|e*r | h_exons_3 | - | mammalian_rnd_2 | 1.962500 |
| D\|c*r | amniotic CNEs | - | ucnes95-100_rnd | 1.961900 |
| D\|e*r | h_exons_3 | - | h_exons_rnd_3 | 1.953700 |
| D\|r*e | amniotic_rnd | - | h_exons_3 | 1.952100 |
| D\|r*e | cnes80-85_rnd | - | h_exons_3 | 1.950900 |
| D\|c*r | amniotic CNEs | - | amniotic_rnd | 1.946000 |
| D\|c*r | amniotic CNEs | - | mammalian_rnd_1 | 1.944000 |
| D\|c*r | amniotic CNEs | - | cnes75-80_rnd | 1.938000 |

```
D|c*r |   amniotic CNEs     -  h_exons_rnd_2     |      1.937300
D|e*r |   h_exons_3         -  h_exons_rnd_1     |      1.937000
D|c*r |   amniotic CNEs     -  cnes80-85_rnd     |      1.932600
D|c*r |   amniotic CNEs     -  mammalian_rnd_2   |      1.930800
D|c*r |   cnes85-90         -  cnes85-90_rnd     |      1.927700
D|c*r |   amniotic CNEs     -  h_exons_rnd_3     |      1.912200
D|c*e |   cnes80-85         -  h_exons_1         |      1.911700
D|c*r |   cnes85-90         -  ucnes95-100_rnd   |      1.909700
D|c*e |   cnes80-85         -  h_exons_3         |      1.906700
D|c*r |   cnes85-90         -  mammalian_rnd_1   |      1.904600
D|r*c |   cnes75-80_rnd     -  cnes85-90         |      1.891800
D|r*c |   amniotic_rnd      -  cnes85-90         |      1.891600
D|r*c |   cnes80-85_rnd     -  cnes85-90         |      1.885000
D|c*r |   amniotic CNEs     -  cnes90-95_rnd     |      1.884700
D|c*r |   cnes85-90         -  h_exons_rnd_2     |      1.883300
D|c*r |   amniotic CNEs     -  h_exons_rnd_1     |      1.882900
D|r*e |   cnes90-95_rnd     -  h_exons_3         |      1.880200
D|c*r |   cnes85-90         -  mammalian_rnd_2   |      1.879200
D|c*r |   cnes85-90         -  h_exons_rnd_3     |      1.861600
D|c*r |   cnes85-90         -  cnes90-95_rnd     |      1.840100
D|c*r |   cnes85-90         -  h_exons_rnd_1     |      1.832500
D|c*e |   cnes75-80         -  h_exons_1         |      1.827300
D|r*c |   cnes90-95_rnd     -  mammalian_CNEs_2  |      1.813400
D|r*c |   amniotic_rnd      -  mammalian_CNEs_2  |      1.809500
D|c*e |   cnes75-80         -  h_exons_3         |      1.804500
D|r*c |   cnes85-90_rnd     -  mammalian_CNEs_2  |      1.796400
D|c*r |   mammalian_CNEs_2  -  ucnes95-100_rnd   |      1.794000
D|c*r |   mammalian_CNEs_2  -  mammalian_rnd_1   |      1.780900
D|c*r |   mammalian_CNEs_2  -  mammalian_rnd_2   |      1.764100
D|r*c |   cnes80-85_rnd     -  mammalian_CNEs_2  |      1.754900
D|r*c |   cnes75-80_rnd     -  mammalian_CNEs_2  |      1.719700
D|c*r |   cnes80-85         -  cnes85-90_rnd     |      1.713500
D|r*c |   h_exons_rnd_2     -  mammalian_CNEs_2  |      1.709800
D|r*c |   h_exons_rnd_3     -  mammalian_CNEs_2  |      1.704900
D|c*r |   cnes80-85         -  mammalian_rnd_1   |      1.691600
D|c*r |   cnes80-85         -  h_exons_rnd_2     |      1.680100
D|r*c |   cnes75-80_rnd     -  cnes80-85         |      1.675200
D|c*r |   cnes80-85         -  ucnes95-100_rnd   |      1.672900
D|c*r |   cnes80-85         -  cnes80-85_rnd     |      1.671000
D|r*c |   amniotic_rnd      -  cnes80-85         |      1.670200
D|c*r |   cnes80-85         -  mammalian_rnd_2   |      1.661800
D|r*c |   h_exons_rnd_1     -  mammalian_CNEs_2  |      1.659000
D|c*r |   cnes80-85         -  h_exons_rnd_3     |      1.654800
D|c*r |   cnes80-85         -  h_exons_rnd_1     |      1.636300
D|r*c |   cnes85-90_rnd     -  mammalian_CNEs_1  |      1.623600
D|c*r |   cnes80-85         -  cnes90-95_rnd     |      1.617100
D|c*r |   mammalian_CNEs_1  -  ucnes95-100_rnd   |      1.595200
D|r*c |   amniotic_rnd      -  mammalian_CNEs_1  |      1.593700
```

```
D|c*r |   mammalian_CNEs_1  -  mammalian_rnd_1    |      1.588900
D|r*c |   cnes90-95_rnd     -  mammalian_CNEs_1   |      1.588800
D|c*r |   cnes75-80         -  cnes85-90_rnd      |      1.577900
D|c*r |   mammalian_CNEs_1  -  mammalian_rnd_2    |      1.574300
D|r*c |   cnes80-85_rnd     -  mammalian_CNEs_1   |      1.556900
D|r*c |   cnes75-80_rnd     -  mammalian_CNEs_1   |      1.555500
D|r*c |   h_exons_rnd_2     -  mammalian_CNEs_1   |      1.555000
D|c*r |   cnes75-80         -  h_exons_rnd_2      |      1.540300
D|c*r |   cnes75-80         -  mammalian_rnd_1    |      1.533600
D|r*c |   h_exons_rnd_3     -  mammalian_CNEs_1   |      1.528700
D|c*r |   cnes75-80         -  cnes80-85_rnd      |      1.524200
D|c*r |   cnes75-80         -  mammalian_rnd_2    |      1.521000
D|c*r |   cnes75-80         -  h_exons_rnd_3      |      1.516200
D|c*r |   cnes75-80         -  cnes75-80_rnd      |      1.515800
D|c*r |   cnes75-80         -  h_exons_rnd_1      |      1.504700
D|c*r |   cnes75-80         -  ucnes95-100_rnd    |      1.504700
S|c*c |   mammalian_CNEs_2  -  ucnes95-100        |      1.504500 #01
D|r*c |   amniotic_rnd      -  cnes75-80          |      1.501000
D|r*c |   h_exons_rnd_1     -  mammalian_CNEs_1   |      1.494800
D|c*r |   cnes75-80         -  cnes90-95_rnd      |      1.459700
===============================================================
S|c*c |   cnes90-95         -  mammalian_CNEs_2   |      1.365901
S|c*c |   cnes75-80         -  mammalian_CNEs_2   |      1.298900
S|c*c |   cnes80-85         -  mammalian_CNEs_2   |      1.255500
S|c*c |   mammalian_CNEs_1  -  ucnes95-100        |      1.246100
S|c*c |   cnes85-90         -  mammalian_CNEs_2   |      1.240500
S|c*c |   amniotic CNEs     -  mammalian_CNEs_2   |      1.185100
S|c*c |   cnes90-95         -  mammalian_CNEs_1   |      1.074501
S|c*c |   cnes75-80         -  ucnes95-100        |      0.939600
S|c*c |   amniotic CNEs     -  mammalian_CNEs_1   |      0.924500
S|c*c |   cnes85-90         -  mammalian_CNEs_1   |      0.924100
S|c*c |   cnes75-80         -  mammalian_CNEs_1   |      0.916100
S|c*c |   cnes80-85         -  mammalian_CNEs_1   |      0.899300
S|c*c |   cnes80-85         -  ucnes95-100        |      0.732000
S|c*c |   cnes75-80         -  cnes90-95          |      0.716000
S|c*c |   amniotic CNEs     -  cnes75-80          |      0.688200
S|c*c |   amniotic CNEs     -  cnes80-85          |      0.534600
S|c*c |   cnes80-85         -  cnes90-95          |      0.530000
S|c*c |   cnes85-90         -  ucnes95-100        |      0.512000
S|c*c |   cnes75-80         -  cnes85-90          |      0.502000
S|c*c |   amniotic CNEs     -  ucnes95-100        |      0.490200
S|c*c |   mammalian_CNEs_1  -  mammalian_CNEs_2   |      0.485000
S|e*e |   h_exons_2         -  h_exons_3          |      0.397100
S|c*c |   amniotic CNEs     -  cnes90-95          |      0.345600
S|e*e |   h_exons_1         -  h_exons_2          |      0.337700
S|c*c |   amniotic CNEs     -  cnes85-90          |      0.337400
S|c*c |   cnes90-95         -  ucnes95-100        |      0.321999
S|c*c |   cnes85-90         -  cnes90-95          |      0.310400
```

| | | | | | | |
|---|---|---|---|---|---|---|
| S\|c*c | \| | cnes80-85 | - | cnes85-90 | \| | 0.307800 |
| S\|c*c | \| | cnes75-80 | - | cnes80-85 | \| | 0.275600 |
| S\|r*r | \| | cnes85-90_rnd | - | cnes90-95_rnd | \| | 0.222200 |
| S\|r*r | \| | cnes90-95_rnd | - | h_exons_rnd_1 | \| | 0.206000 |
| S\|r*r | \| | cnes85-90_rnd | - | h_exons_rnd_1 | \| | 0.192800 |
| S\|r*r | \| | cnes85-90_rnd | - | ucnes95-100_rnd | \| | 0.192201 |
| S\|r*r | \| | cnes90-95_rnd | - | h_exons_rnd_2 | \| | 0.188600 |
| S\|r*r | \| | amniotic_rnd | - | h_exons_rnd_1 | \| | 0.184500 |
| S\|r*r | \| | h_exons_rnd_2 | - | ucnes95-100_rnd | \| | 0.183600 |
| S\|r*r | \| | h_exons_rnd_1 | - | ucnes95-100_rnd | \| | 0.182600 |
| S\|r*r | \| | cnes90-95_rnd | - | ucnes95-100_rnd | \| | 0.179800 |
| S\|r*r | \| | cnes80-85_rnd | - | cnes85-90_rnd | \| | 0.178100 |
| S\|r*r | \| | cnes90-95_rnd | - | h_exons_rnd_3 | \| | 0.175700 |
| S\|e*e | \| | h_exons_1 | - | h_exons_3 | \| | 0.173400 |
| S\|r*r | \| | cnes80-85_rnd | - | ucnes95-100_rnd | \| | 0.169300 |
| S\|r*r | \| | cnes75-80_rnd | - | cnes90-95_rnd | \| | 0.168300 |
| S\|r*r | \| | cnes75-80_rnd | - | cnes85-90_rnd | \| | 0.165900 |
| S\|r*r | \| | cnes85-90_rnd | - | h_exons_rnd_3 | \| | 0.165300 |
| S\|r*r | \| | cnes85-90_rnd | - | h_exons_rnd_2 | \| | 0.155200 |
| S\|r*r | \| | cnes90-95_rnd | - | mammalian_rnd_1 | \| | 0.154300 |
| S\|r*r | \| | amniotic_rnd | - | h_exons_rnd_2 | \| | 0.153500 |
| S\|r*r | \| | cnes80-85_rnd | - | cnes90-95_rnd | \| | 0.153500 |
| S\|r*r | \| | h_exons_rnd_3 | - | ucnes95-100_rnd | \| | 0.152700 |
| S\|r*r | \| | cnes90-95_rnd | - | mammalian_rnd_2 | \| | 0.150900 |
| S\|r*r | \| | amniotic_rnd | - | cnes85-90_rnd | \| | 0.145700 |
| S\|r*r | \| | h_exons_rnd_1 | - | mammalian_rnd_1 | \| | 0.143300 |
| S\|r*r | \| | amniotic_rnd | - | h_exons_rnd_3 | \| | 0.140600 |
| S\|r*r | \| | cnes80-85_rnd | - | h_exons_rnd_1 | \| | 0.140500 |
| S\|r*r | \| | amniotic_rnd | - | cnes80-85_rnd | \| | 0.138200 |
| S\|r*r | \| | mammalian_rnd_1 | - | ucnes95-100_rnd | \| | 0.135100 |
| S\|r*r | \| | cnes75-80_rnd | - | ucnes95-100_rnd | \| | 0.134300 |
| S\|r*r | \| | cnes85-90_rnd | - | mammalian_rnd_2 | \| | 0.134300 |
| S\|r*r | \| | cnes80-85_rnd | - | h_exons_rnd_2 | \| | 0.130500 |
| S\|r*r | \| | amniotic_rnd | - | cnes90-95_rnd | \| | 0.129900 |
| S\|r*r | \| | cnes75-80_rnd | - | cnes80-85_rnd | \| | 0.129800 |
| S\|r*r | \| | cnes85-90_rnd | - | mammalian_rnd_1 | \| | 0.129100 |
| S\|r*r | \| | mammalian_rnd_2 | - | ucnes95-100_rnd | \| | 0.127300 |
| S\|r*r | \| | cnes80-85_rnd | - | mammalian_rnd_1 | \| | 0.125800 |
| S\|r*r | \| | h_exons_rnd_1 | - | mammalian_rnd_2 | \| | 0.124900 |
| S\|r*r | \| | amniotic_rnd | - | cnes75-80_rnd | \| | 0.124400 |
| S\|r*r | \| | amniotic_rnd | - | ucnes95-100_rnd | \| | 0.123300 |
| S\|r*r | \| | cnes80-85_rnd | - | h_exons_rnd_3 | \| | 0.119400 |
| S\|r*r | \| | cnes80-85_rnd | - | mammalian_rnd_2 | \| | 0.109400 |
| S\|r*r | \| | h_exons_rnd_2 | - | mammalian_rnd_1 | \| | 0.103100 |
| S\|r*r | \| | cnes75-80_rnd | - | h_exons_rnd_1 | \| | 0.100300 |
| S\|r*r | \| | amniotic_rnd | - | mammalian_rnd_1 | \| | 0.099200 |
| S\|r*r | \| | h_exons_rnd_2 | - | mammalian_rnd_2 | \| | 0.097900 |
| S\|r*r | \| | amniotic_rnd | - | mammalian_rnd_2 | \| | 0.092800 |

```
S|r*r |   h_exons_rnd_3     -  mammalian_rnd_1   |      0.091400
S|r*r |   cnes75-80_rnd     -  h_exons_rnd_2     |      0.090100
S|r*r |   cnes75-80_rnd     -  mammalian_rnd_1   |      0.081800
S|r*r |   cnes75-80_rnd     -  mammalian_rnd_2   |      0.077200
S|r*r |   h_exons_rnd_1     -  h_exons_rnd_2     |      0.074800
S|r*r |   h_exons_rnd_1     -  h_exons_rnd_3     |      0.072100
S|r*r |   cnes75-80_rnd     -  h_exons_rnd_3     |      0.066600
S|r*r |   h_exons_rnd_3     -  mammalian_rnd_2   |      0.065400
S|r*r |   mammalian_rnd_1   -  mammalian_rnd_2   |      0.064800
S|r*r |   h_exons_rnd_2     -  h_exons_rnd_3     |      0.062700

######################################################################
```

## DISTANCES FOR ALL PAIRS OF DATASETS FOR TETRAPLETS

THERE ARE ONLY 3/231 DEVIATIONS FROM THE PERFECT FRACTIONATION BETWEEN COMPARISONS OF 'DIFFERENT (D)' (c*e OR e*c, r*c OR c*r, e*r OR r*e) (UPPER LAYER, LONG Delta-DISTANCE) AND OF 'SIMILAR (S)' (c*c, e*e, r*r) (LOWER LAYER, SHORT Delta-DISTANCE) DATASETS. c, e, r STAND FOR CNEs, EXONS AND RANDOMLY CHOSEN SEGMENTS (SURROGATES). ALL 3 DEVIATIONS INVOLVE 'mammalian_CNEs'

```
D-OR-S|DATASETS WHOSE Delta-DISTANCE IS COMPUTED |Delta-DISTANCE
----------------------------------------------------------------
D|e*c |   h_exons_2         -  ucnes95-100       |     13.491701
D|c*e |   cnes90-95         -  h_exons_2         |     12.900303
D|c*e |   amniotic CNEs     -  h_exons_2         |     12.649701
D|e*c |   h_exons_2         -  mammalian_CNEs_1  |     12.503096
D|e*r |   h_exons_2         -  ucnes95-100_rnd   |     12.202804
D|r*e |   cnes90-95_rnd     -  h_exons_2         |     12.162500
D|c*e |   cnes85-90         -  h_exons_2         |     12.084699
D|r*e |   cnes75-80_rnd     -  h_exons_2         |     11.959101
D|r*e |   cnes80-85_rnd     -  h_exons_2         |     11.939701
D|e*r |   h_exons_2         -  mammalian_rnd_1   |     11.922795
D|r*e |   cnes85-90_rnd     -  h_exons_2         |     11.915398
D|e*c |   h_exons_1         -  ucnes95-100       |     11.867500
D|e*r |   h_exons_2         -  mammalian_rnd_2   |     11.783498
D|r*e |   amniotic_rnd      -  h_exons_2         |     11.679600
D|e*r |   h_exons_2         -  h_exons_rnd_3     |     11.635201
D|e*c |   h_exons_3         -  ucnes95-100       |     11.633998
D|e*r |   h_exons_2         -  h_exons_rnd_1     |     11.603602
D|e*r |   h_exons_2         -  h_exons_rnd_2     |     11.590702
D|e*c |   h_exons_2         -  mammalian_CNEs_2  |     11.368198
D|c*e |   cnes90-95         -  h_exons_1         |     11.225499
D|c*e |   amniotic CNEs     -  h_exons_1         |     11.054498
```

| | | | | | |
|---|---|---|---|---|---|
| D\|c*e | cnes90-95 | - | h_exons_3 | | 11.027200 |
| D\|r*e | cnes90-95_rnd | - | h_exons_1 | | 10.949502 |
| D\|c*e | cnes80-85 | - | h_exons_2 | | 10.944996 |
| D\|e*c | h_exons_1 | - | mammalian_CNEs_1 | | 10.934504 |
| D\|e*r | h_exons_1 | - | ucnes95-100_rnd | | 10.902199 |
| D\|r*c | cnes90-95_rnd | - | ucnes95-100 | | 10.863997 |
| D\|c*e | amniotic CNEs | - | h_exons_3 | | 10.733403 |
| D\|r*e | cnes75-80_rnd | - | h_exons_1 | | 10.688700 |
| D\|r*e | cnes85-90_rnd | - | h_exons_1 | | 10.654795 |
| D\|e*r | h_exons_1 | - | mammalian_rnd_1 | | 10.653603 |
| D\|r*e | cnes80-85_rnd | - | h_exons_1 | | 10.643101 |
| D\|r*c | cnes75-80_rnd | - | ucnes95-100 | | 10.615998 |
| D\|r*c | h_exons_rnd_1 | - | ucnes95-100 | | 10.612302 |
| D\|r*c | h_exons_rnd_3 | - | ucnes95-100 | | 10.611503 |
| D\|r*c | h_exons_rnd_2 | - | ucnes95-100 | | 10.597801 |
| D\|c*e | cnes75-80 | - | h_exons_2 | | 10.583197 |
| D\|r*c | cnes85-90_rnd | - | ucnes95-100 | | 10.567500 |
| D\|r*e | cnes90-95_rnd | - | h_exons_3 | | 10.564603 |
| D\|r*c | mammalian_rnd_1 | - | ucnes95-100 | | 10.536901 |
| D\|c*r | ucnes95-100 | - | ucnes95-100_rnd | | 10.532899 |
| D\|e*r | h_exons_3 | - | ucnes95-100_rnd | | 10.530901 |
| D\|r*c | mammalian_rnd_2 | - | ucnes95-100 | | 10.498202 |
| D\|e*r | h_exons_1 | - | mammalian_rnd_2 | | 10.494899 |
| D\|e*c | h_exons_3 | - | mammalian_CNEs_1 | | 10.443401 |
| D\|c*e | cnes85-90 | - | h_exons_1 | | 10.417701 |
| D\|r*e | amniotic_rnd | - | h_exons_1 | | 10.392601 |
| D\|r*c | cnes80-85_rnd | - | ucnes95-100 | | 10.386200 |
| D\|e*r | h_exons_1 | - | h_exons_rnd_2 | | 10.369102 |
| D\|r*c | amniotic_rnd | - | ucnes95-100 | | 10.362899 |
| D\|e*r | h_exons_1 | - | h_exons_rnd_1 | | 10.355400 |
| D\|e*r | h_exons_1 | - | h_exons_rnd_3 | | 10.332996 |
| D\|r*e | cnes75-80_rnd | - | h_exons_3 | | 10.313003 |
| D\|r*e | cnes85-90_rnd | - | h_exons_3 | | 10.289702 |
| D\|e*r | h_exons_3 | - | mammalian_rnd_1 | | 10.287700 |
| D\|r*e | cnes80-85_rnd | - | h_exons_3 | | 10.232396 |
| D\|e*r | h_exons_3 | - | mammalian_rnd_2 | | 10.117804 |
| D\|r*e | amniotic_rnd | - | h_exons_3 | | 10.046900 |
| D\|c*e | cnes85-90 | - | h_exons_3 | | 10.018403 |
| D\|e*r | h_exons_3 | - | h_exons_rnd_2 | | 10.010196 |
| D\|e*r | h_exons_3 | - | h_exons_rnd_1 | | 10.003502 |
| D\|e*r | h_exons_3 | - | h_exons_rnd_3 | | 9.991497 |
| D\|e*c | h_exons_1 | - | mammalian_CNEs_2 | | 9.946002 |
| D\|c*r | cnes90-95 | - | cnes90-95_rnd | | 9.559799 |
| D\|e*c | h_exons_3 | - | mammalian_CNEs_2 | | 9.412700 |
| D\|r*c | cnes85-90_rnd | - | cnes90-95 | | 9.305898 |
| D\|c*e | cnes80-85 | - | h_exons_1 | | 9.299000 |
| D\|c*r | cnes90-95 | - | h_exons_rnd_3 | | 9.245103 |
| D\|c*r | cnes90-95 | - | h_exons_rnd_2 | | 9.235598 |

```
D|r*c |   cnes75-80_rnd      -  cnes90-95          |      9.231798
D|c*r |   cnes90-95          -  h_exons_rnd_1      |      9.206496
D|c*r |   cnes90-95          -  mammalian_rnd_1    |      9.180698
D|c*r |   cnes90-95          -  ucnes95-100_rnd    |      9.143301
D|c*r |   cnes90-95          -  mammalian_rnd_2    |      9.120400
D|r*c |   cnes80-85_rnd      -  cnes90-95          |      9.064798
D|c*r |   amniotic CNEs      -  cnes90-95_rnd      |      9.063601
D|r*c |   amniotic_rnd       -  cnes90-95          |      9.031696
D|c*e |   cnes80-85          -  h_exons_3          |      9.001101
D|c*e |   cnes75-80          -  h_exons_1          |      8.902399
D|c*r |   amniotic CNEs      -  cnes85-90_rnd      |      8.811899
D|c*r |   amniotic CNEs      -  h_exons_rnd_2      |      8.778997
D|c*r |   amniotic CNEs      -  cnes75-80_rnd      |      8.778799
D|c*r |   amniotic CNEs      -  h_exons_rnd_3      |      8.771701
D|c*r |   amniotic CNEs      -  ucnes95-100_rnd    |      8.758301
D|c*r |   amniotic CNEs      -  h_exons_rnd_1      |      8.732099
D|c*r |   amniotic CNEs      -  mammalian_rnd_1    |      8.714498
D|c*r |   amniotic CNEs      -  mammalian_rnd_2    |      8.647999
D|c*r |   amniotic CNEs      -  cnes80-85_rnd      |      8.545600
D|c*e |   cnes75-80          -  h_exons_3          |      8.519299
D|c*r |   amniotic CNEs      -  amniotic_rnd       |      8.489099
S|c*c |   mammalian_CNEs_2   -  ucnes95-100        |      8.455698 #01
D|c*r |   cnes85-90          -  cnes90-95_rnd      |      8.379200
D|r*c |   cnes85-90_rnd      -  mammalian_CNEs_1   |      8.286900
D|c*r |   cnes85-90          -  cnes85-90_rnd      |      8.278500
D|c*r |   cnes85-90          -  ucnes95-100_rnd    |      8.078702
D|c*r |   mammalian_CNEs_1   -  ucnes95-100_rnd    |      8.076502
D|c*r |   cnes85-90          -  h_exons_rnd_2      |      8.063801
D|c*r |   cnes85-90          -  mammalian_rnd_1    |      8.053700
D|r*c |   cnes75-80_rnd      -  cnes85-90          |      8.048199
D|c*r |   cnes85-90          -  h_exons_rnd_3      |      8.037902
D|c*r |   cnes85-90          -  h_exons_rnd_1      |      7.998702
S|c*c |   mammalian_CNEs_1   -  ucnes95-100        |      7.976800 #02
D|c*r |   cnes85-90          -  mammalian_rnd_2    |      7.961399
D|r*c |   cnes80-85_rnd      -  cnes85-90          |      7.918600
D|r*c |   cnes90-95_rnd      -  mammalian_CNEs_1   |      7.881001
D|r*c |   amniotic_rnd       -  cnes85-90          |      7.874301
D|c*r |   mammalian_CNEs_1   -  mammalian_rnd_1    |      7.851901
D|r*c |   cnes85-90_rnd      -  mammalian_CNEs_2   |      7.796599
D|r*c |   cnes75-80_rnd      -  mammalian_CNEs_1   |      7.780199
D|c*r |   mammalian_CNEs_2   -  ucnes95-100_rnd    |      7.764201
D|c*r |   cnes80-85          -  cnes85-90_rnd      |      7.734199
D|c*r |   cnes80-85          -  cnes90-95_rnd      |      7.706499
D|r*c |   h_exons_rnd_2      -  mammalian_CNEs_1   |      7.690799
D|r*c |   cnes80-85_rnd      -  mammalian_CNEs_1   |      7.657802
D|c*r |   cnes80-85          -  mammalian_rnd_1    |      7.640595
D|c*r |   cnes80-85          -  ucnes95-100_rnd    |      7.640199
D|r*c |   amniotic_rnd       -  mammalian_CNEs_1   |      7.606700
```

```
D|c*r |   mammalian_CNEs_1   -  mammalian_rnd_2    |      7.592800
D|r*c |   cnes75-80_rnd      -  cnes80-85          |      7.584499
D|r*c |   h_exons_rnd_3      -  mammalian_CNEs_1   |      7.491899
D|c*r |   cnes80-85          -  h_exons_rnd_2      |      7.467499
D|c*r |   mammalian_CNEs_2   -  mammalian_rnd_1    |      7.422200
D|r*c |   cnes90-95_rnd      -  mammalian_CNEs_2   |      7.402299
D|c*r |   cnes80-85          -  mammalian_rnd_2    |      7.387499
D|c*r |   cnes80-85          -  h_exons_rnd_3      |      7.337199
D|c*r |   cnes80-85          -  cnes80-85_rnd      |      7.311503
D|r*c |   cnes75-80_rnd      -  mammalian_CNEs_2   |      7.306097
D|c*r |   cnes80-85          -  h_exons_rnd_1      |      7.286399
D|r*c |   h_exons_rnd_1      -  mammalian_CNEs_1   |      7.236900
D|c*r |   cnes75-80          -  cnes85-90_rnd      |      7.215001
D|r*c |   cnes80-85_rnd      -  mammalian_CNEs_2   |      7.196699
D|r*c |   amniotic_rnd       -  mammalian_CNEs_2   |      7.181998
D|r*c |   amniotic_rnd       -  cnes80-85          |      7.174398
D|c*r |   cnes75-80          -  cnes90-95_rnd      |      7.171701
D|c*r |   mammalian_CNEs_2   -  mammalian_rnd_2    |      7.157701
D|r*c |   h_exons_rnd_2      -  mammalian_CNEs_2   |      7.088697
D|c*r |   cnes75-80          -  ucnes95-100_rnd    |      7.031400
S|c*c |   cnes90-95          -  mammalian_CNEs_2   |      6.991899 #03
D|c*r |   cnes75-80          -  mammalian_rnd_1    |      6.989199
D|r*c |   h_exons_rnd_3      -  mammalian_CNEs_2   |      6.968000
D|c*r |   cnes75-80          -  cnes75-80_rnd      |      6.963499
D|c*r |   cnes75-80          -  h_exons_rnd_2      |      6.952701
D|c*r |   cnes75-80          -  h_exons_rnd_1      |      6.896601
D|c*r |   cnes75-80          -  h_exons_rnd_3      |      6.880398
D|c*r |   cnes75-80          -  mammalian_rnd_2    |      6.849699
D|c*r |   cnes75-80          -  cnes80-85_rnd      |      6.751299
D|r*c |   h_exons_rnd_1      -  mammalian_CNEs_2   |      6.742201
D|r*c |   amniotic_rnd       -  cnes75-80          |      6.655598
=================================================================
S|c*c |   cnes90-95          -  mammalian_CNEs_1   |      6.459802
S|c*c |   amniotic CNEs      -  mammalian_CNEs_2   |      5.999900
S|c*c |   cnes85-90          -  mammalian_CNEs_2   |      5.816701
S|c*c |   cnes75-80          -  ucnes95-100        |      5.592101
S|c*c |   cnes85-90          -  mammalian_CNEs_1   |      5.514001
S|c*c |   amniotic CNEs      -  mammalian_CNEs_1   |      5.368398
S|c*c |   cnes80-85          -  mammalian_CNEs_2   |      5.088999
S|c*c |   cnes80-85          -  ucnes95-100        |      4.916700
S|c*c |   cnes80-85          -  mammalian_CNEs_1   |      4.887898
S|c*c |   cnes75-80          -  mammalian_CNEs_2   |      4.846602
S|c*c |   cnes75-80          -  mammalian_CNEs_1   |      4.752300
S|c*c |   cnes75-80          -  cnes90-95          |      4.134100
S|c*c |   cnes85-90          -  ucnes95-100        |      3.710800
S|c*c |   amniotic CNEs      -  cnes75-80          |      3.464299
S|c*c |   cnes80-85          -  cnes90-95          |      3.456101
S|c*c |   amniotic CNEs      -  ucnes95-100        |      3.170800
```

| | | | | |
|---|---|---|---|---|
| S\|c*c | amniotic CNEs | - | cnes80-85 | 2.982500 |
| S\|e*e | h_exons_2 | - | h_exons_3 | 2.855900 |
| S\|c*c | cnes75-80 | - | cnes85-90 | 2.719100 |
| S\|c*c | cnes90-95 | - | ucnes95-100 | 2.467800 |
| S\|e*e | h_exons_1 | - | h_exons_2 | 2.379600 |
| S\|c*c | cnes80-85 | - | cnes85-90 | 2.346300 |
| S\|c*c | cnes85-90 | - | cnes90-95 | 2.344399 |
| S\|c*c | amniotic CNEs | - | cnes85-90 | 2.219199 |
| S\|c*c | mammalian_CNEs_1 | - | mammalian_CNEs_2 | 2.104100 |
| S\|c*c | amniotic CNEs | - | cnes90-95 | 1.916200 |
| S\|r*r | cnes85-90_rnd | - | cnes90-95_rnd | 1.739099 |
| S\|r*r | cnes90-95_rnd | - | ucnes95-100_rnd | 1.661700 |
| S\|c*c | cnes75-80 | - | cnes80-85 | 1.659399 |
| S\|r*r | h_exons_rnd_1 | - | ucnes95-100_rnd | 1.624999 |
| S\|r*r | cnes85-90_rnd | - | h_exons_rnd_1 | 1.616799 |
| S\|r*r | cnes85-90_rnd | - | ucnes95-100_rnd | 1.580800 |
| S\|r*r | cnes75-80_rnd | - | ucnes95-100_rnd | 1.575699 |
| S\|r*r | cnes75-80_rnd | - | cnes90-95_rnd | 1.489401 |
| S\|r*r | amniotic_rnd | - | cnes90-95_rnd | 1.489100 |
| S\|r*r | h_exons_rnd_3 | - | ucnes95-100_rnd | 1.486598 |
| S\|r*r | cnes90-95_rnd | - | h_exons_rnd_2 | 1.431200 |
| S\|r*r | cnes85-90_rnd | - | h_exons_rnd_3 | 1.426599 |
| S\|r*r | cnes90-95_rnd | - | h_exons_rnd_1 | 1.407500 |
| S\|r*r | amniotic_rnd | - | ucnes95-100_rnd | 1.396200 |
| S\|r*r | cnes90-95_rnd | - | h_exons_rnd_3 | 1.365100 |
| S\|r*r | cnes80-85_rnd | - | cnes90-95_rnd | 1.344801 |
| S\|r*r | mammalian_rnd_2 | - | ucnes95-100_rnd | 1.344100 |
| S\|r*r | h_exons_rnd_2 | - | ucnes95-100_rnd | 1.341300 |
| S\|r*r | cnes80-85_rnd | - | ucnes95-100_rnd | 1.333899 |
| S\|r*r | cnes90-95_rnd | - | mammalian_rnd_1 | 1.330901 |
| S\|r*r | cnes75-80_rnd | - | h_exons_rnd_1 | 1.274299 |
| S\|r*r | amniotic_rnd | - | cnes85-90_rnd | 1.236200 |
| S\|r*r | cnes90-95_rnd | - | mammalian_rnd_2 | 1.212800 |
| S\|r*r | cnes85-90_rnd | - | mammalian_rnd_2 | 1.195899 |
| S\|r*r | mammalian_rnd_1 | - | ucnes95-100_rnd | 1.190400 |
| S\|r*r | cnes80-85_rnd | - | cnes85-90_rnd | 1.185500 |
| S\|r*r | cnes85-90_rnd | - | h_exons_rnd_2 | 1.181300 |
| S\|r*r | cnes75-80_rnd | - | cnes85-90_rnd | 1.179500 |
| S\|r*r | cnes80-85_rnd | - | h_exons_rnd_1 | 1.173100 |
| S\|e*e | h_exons_1 | - | h_exons_3 | 1.134700 |
| S\|r*r | cnes75-80_rnd | - | cnes80-85_rnd | 1.120201 |
| S\|r*r | cnes85-90_rnd | - | mammalian_rnd_1 | 1.091000 |
| S\|r*r | h_exons_rnd_1 | - | mammalian_rnd_1 | 1.071000 |
| S\|r*r | cnes80-85_rnd | - | h_exons_rnd_3 | 1.070700 |
| S\|r*r | cnes75-80_rnd | - | h_exons_rnd_3 | 1.029900 |
| S\|r*r | amniotic_rnd | - | cnes75-80_rnd | 0.998500 |
| S\|r*r | amniotic_rnd | - | h_exons_rnd_1 | 0.953800 |
| S\|r*r | cnes80-85_rnd | - | h_exons_rnd_2 | 0.941600 |

```
S|r*r |   cnes75-80_rnd      -  h_exons_rnd_2     |      0.926400
S|r*r |   amniotic_rnd       -  cnes80-85_rnd     |      0.895901
S|r*r |   cnes80-85_rnd      -  mammalian_rnd_1   |      0.861900
S|r*r |   cnes75-80_rnd      -  mammalian_rnd_2   |      0.861401
S|r*r |   cnes80-85_rnd      -  mammalian_rnd_2   |      0.859000
S|r*r |   cnes75-80_rnd      -  mammalian_rnd_1   |      0.852301
S|r*r |   h_exons_rnd_1      -  mammalian_rnd_2   |      0.842300
S|r*r |   amniotic_rnd       -  mammalian_rnd_1   |      0.838801
S|r*r |   h_exons_rnd_1      -  h_exons_rnd_2     |      0.830300
S|r*r |   h_exons_rnd_3      -  mammalian_rnd_1   |      0.793600
S|r*r |   amniotic_rnd       -  h_exons_rnd_3     |      0.791800
S|r*r |   amniotic_rnd       -  h_exons_rnd_2     |      0.729900
S|r*r |   h_exons_rnd_2      -  mammalian_rnd_1   |      0.674099
S|r*r |   amniotic_rnd       -  mammalian_rnd_2   |      0.638900
S|r*r |   h_exons_rnd_1      -  h_exons_rnd_3     |      0.631400
S|r*r |   mammalian_rnd_1    -  mammalian_rnd_2   |      0.630901
S|r*r |   h_exons_rnd_2      -  h_exons_rnd_3     |      0.626300
S|r*r |   h_exons_rnd_2      -  mammalian_rnd_2   |      0.619800
S|r*r |   h_exons_rnd_3      -  mammalian_rnd_2   |      0.618700

######################################################################
```

## DISTANCES FOR ALL PAIRS OF DATASETS FOR PENTAPLETS

THERE ARE ONLY 4/231 DEVIATIONS FROM THE PERFECT FRACTIONATION BETWEEN COMPARISONS OF 'DIFFERENT (D)' (c*e OR e*c, r*c OR c*r, e*r OR r*e) (UPPER LAYER, LONG Delta-DISTANCE) AND OF 'SIMILAR (S)' (c*c, e*e, r*r) (LOWER LAYER, SHORT Delta-DISTANCE) DATASETS. c, e, r STAND FOR CNEs, EXONS AND RANDOMLY CHOSEN SEGMENTS (SURROGATES). ALL 4 DEVIATIONS INVOLVE 'mammalian_CNEs'

```
D-OR-S|DATASETS WHOSE Delta-DISTANCE IS COMPUTED |Delta-DISTANCE
----------------------------------------------------------------
D|e*r |   h_exons_2          -  ucnes95-100_rnd   |     50.273968
D|r*e |   cnes90-95_rnd      -  h_exons_2         |     49.863998
D|r*e |   cnes85-90_rnd      -  h_exons_2         |     49.566284
D|e*r |   h_exons_2          -  mammalian_rnd_1   |     49.118008
D|r*e |   amniotic_rnd       -  h_exons_2         |     48.904003
D|r*e |   cnes75-80_rnd      -  h_exons_2         |     48.357101
D|r*e |   cnes80-85_rnd      -  h_exons_2         |     48.179588
D|e*r |   h_exons_1          -  ucnes95-100_rnd   |     47.968994
D|e*r |   h_exons_3          -  ucnes95-100_rnd   |     47.956902
D|e*r |   h_exons_2          -  mammalian_rnd_2   |     47.801201
D|e*r |   h_exons_2          -  h_exons_rnd_2     |     47.480698
D|r*e |   cnes85-90_rnd      -  h_exons_1         |     47.043083
```

| | | | | |
|---|---|---|---|---|
| D\|r*e | cnes90-95_rnd | - | h_exons_3 | 46.962303 |
| D\|r*e | cnes90-95_rnd | - | h_exons_1 | 46.931198 |
| D\|r*e | cnes85-90_rnd | - | h_exons_3 | 46.899582 |
| D\|c*r | ucnes95-100 | - | ucnes95-100_rnd | 46.769184 |
| D\|e*r | h_exons_1 | - | mammalian_rnd_1 | 46.624802 |
| D\|e*r | h_exons_2 | - | h_exons_rnd_3 | 46.609688 |
| D\|e*r | h_exons_3 | - | mammalian_rnd_1 | 46.542709 |
| D\|r*e | amniotic_rnd | - | h_exons_1 | 46.502384 |
| D\|r*e | amniotic_rnd | - | h_exons_3 | 46.440918 |
| D\|r*e | cnes75-80_rnd | - | h_exons_1 | 46.286903 |
| D\|r*c | cnes85-90_rnd | - | ucnes95-100 | 46.223907 |
| D\|r*c | cnes90-95_rnd | - | ucnes95-100 | 46.097404 |
| D\|e*r | h_exons_2 | - | h_exons_rnd_1 | 45.937305 |
| D\|r*e | cnes75-80_rnd | - | h_exons_3 | 45.911419 |
| D\|r*c | cnes80-85_rnd | - | ucnes95-100 | 45.902012 |
| D\|r*e | cnes80-85_rnd | - | h_exons_3 | 45.692921 |
| D\|r*e | cnes80-85_rnd | - | h_exons_1 | 45.675610 |
| D\|r*c | amniotic_rnd | - | ucnes95-100 | 45.640205 |
| D\|r*c | mammalian_rnd_1 | - | ucnes95-100 | 45.534580 |
| D\|r*c | cnes75-80_rnd | - | ucnes95-100 | 45.478298 |
| D\|e*r | h_exons_3 | - | mammalian_rnd_2 | 45.273689 |
| D\|r*c | mammalian_rnd_2 | - | ucnes95-100 | 45.265797 |
| D\|e*r | h_exons_1 | - | mammalian_rnd_2 | 45.262413 |
| D\|e*r | h_exons_1 | - | h_exons_rnd_2 | 45.008121 |
| D\|r*c | h_exons_rnd_2 | - | ucnes95-100 | 44.761703 |
| D\|e*r | h_exons_3 | - | h_exons_rnd_2 | 44.688622 |
| D\|c*r | cnes90-95 | - | ucnes95-100_rnd | 44.372700 |
| D\|r*c | h_exons_rnd_3 | - | ucnes95-100 | 44.015480 |
| D\|e*r | h_exons_1 | - | h_exons_rnd_3 | 43.917095 |
| D\|e*r | h_exons_3 | - | h_exons_rnd_3 | 43.742992 |
| D\|e*r | h_exons_1 | - | h_exons_rnd_1 | 43.639297 |
| D\|c*r | cnes90-95 | - | cnes90-95_rnd | 43.478306 |
| D\|e*r | h_exons_3 | - | h_exons_rnd_1 | 43.433620 |
| D\|r*c | cnes85-90_rnd | - | cnes90-95 | 43.191811 |
| D\|r*c | h_exons_rnd_1 | - | ucnes95-100 | 42.847275 |
| D\|c*r | cnes85-90 | - | ucnes95-100_rnd | 42.571609 |
| D\|r*c | amniotic_rnd | - | cnes90-95 | 42.481297 |
| D\|c*r | cnes80-85 | - | ucnes95-100_rnd | 42.417004 |
| D\|c*r | cnes90-95 | - | mammalian_rnd_1 | 42.404881 |
| D\|r*c | cnes75-80_rnd | - | cnes90-95 | 42.376793 |
| D\|r*c | cnes80-85_rnd | - | cnes90-95 | 42.376499 |
| D\|c*r | mammalian_CNEs_1 | - | ucnes95-100_rnd | 41.942585 |
| D\|c*r | amniotic CNEs | - | ucnes95-100_rnd | 41.851681 |
| D\|c*r | cnes90-95 | - | mammalian_rnd_2 | 41.808094 |
| D\|c*r | mammalian_CNEs_2 | - | ucnes95-100_rnd | 41.724197 |
| D\|c*r | cnes80-85 | - | cnes85-90_rnd | 41.723110 |
| D\|c*r | cnes85-90 | - | cnes85-90_rnd | 41.606304 |
| D\|c*r | cnes75-80 | - | ucnes95-100_rnd | 41.571304 |

| | | | | |
|---|---|---|---|---|
| D\|r*c | cnes85-90_rnd | - | mammalian_CNEs_1 | 41.442684 |
| D\|c*r | cnes90-95 | - | h_exons_rnd_2 | 41.394409 |
| D\|c*r | cnes85-90 | - | cnes90-95_rnd | 41.314781 |
| D\|r*c | cnes85-90_rnd | - | mammalian_CNEs_2 | 41.310280 |
| D\|c*r | cnes75-80 | - | cnes85-90_rnd | 41.128002 |
| D\|c*r | amniotic CNEs | - | cnes85-90_rnd | 41.051018 |
| D\|c*r | cnes85-90 | - | mammalian_rnd_1 | 41.033607 |
| D\|r*c | amniotic_rnd | - | cnes85-90 | 41.025185 |
| D\|c*r | cnes80-85 | - | cnes90-95_rnd | 40.959801 |
| D\|r*c | amniotic_rnd | - | cnes80-85 | 40.744602 |
| D\|c*r | cnes90-95 | - | h_exons_rnd_3 | 40.741386 |
| D\|c*r | cnes80-85 | - | mammalian_rnd_1 | 40.650394 |
| D\|c*r | cnes80-85 | - | cnes80-85_rnd | 40.638798 |
| D\|c*r | amniotic CNEs | - | cnes90-95_rnd | 40.610901 |
| D\|r*c | cnes75-80_rnd | - | cnes85-90 | 40.549290 |
| D\|r*c | cnes80-85_rnd | - | cnes85-90 | 40.494598 |
| D\|r*c | cnes90-95_rnd | - | mammalian_CNEs_1 | 40.417015 |
| D\|r*c | cnes75-80_rnd | - | cnes80-85 | 40.278099 |
| D\|r*c | cnes90-95_rnd | - | mammalian_CNEs_2 | 40.250000 |
| D\|c*r | cnes75-80 | - | cnes90-95_rnd | 40.072510 |
| D\|c*r | amniotic CNEs | - | mammalian_rnd_1 | 40.032116 |
| D\|c*r | amniotic CNEs | - | amniotic_rnd | 40.025089 |
| D\|c*r | amniotic CNEs | - | cnes80-85_rnd | 40.021099 |
| D\|r*c | amniotic_rnd | - | mammalian_CNEs_1 | 40.008202 |
| D\|c*r | cnes75-80 | - | mammalian_rnd_1 | 39.993294 |
| D\|c*r | cnes85-90 | - | mammalian_rnd_2 | 39.963604 |
| D\|c*r | mammalian_CNEs_1 | - | mammalian_rnd_1 | 39.873009 |
| D\|c*r | amniotic CNEs | - | cnes75-80_rnd | 39.842400 |
| D\|c*r | cnes80-85 | - | mammalian_rnd_2 | 39.805592 |
| D\|r*c | amniotic_rnd | - | cnes75-80 | 39.782299 |
| D\|c*r | cnes90-95 | - | h_exons_rnd_1 | 39.771397 |
| D\|r*c | amniotic_rnd | - | mammalian_CNEs_2 | 39.757206 |
| D\|c*r | cnes75-80 | - | cnes80-85_rnd | 39.727898 |
| D\|c*r | mammalian_CNEs_2 | - | mammalian_rnd_1 | 39.695602 |
| D\|r*c | cnes80-85_rnd | - | mammalian_CNEs_1 | 39.639797 |
| D\|r*c | cnes75-80_rnd | - | mammalian_CNEs_1 | 39.389496 |
| D\|c*r | amniotic CNEs | - | mammalian_rnd_2 | 39.353107 |
| D\|e*c | h_exons_2 | - | mammalian_CNEs_2 | 39.338985 |
| D\|r*c | cnes80-85_rnd | - | mammalian_CNEs_2 | 39.321407 |
| D\|c*r | cnes85-90 | - | h_exons_rnd_2 | 39.291111 |
| D\|c*r | cnes75-80 | - | cnes75-80_rnd | 39.290005 |
| D\|c*r | mammalian_CNEs_1 | - | mammalian_rnd_2 | 39.259003 |
| D\|r*c | cnes75-80_rnd | - | mammalian_CNEs_2 | 39.172714 |
| D\|c*r | cnes80-85 | - | h_exons_rnd_2 | 39.032513 |
| D\|c*r | cnes75-80 | - | mammalian_rnd_2 | 39.009476 |
| D\|c*r | mammalian_CNEs_2 | - | mammalian_rnd_2 | 38.925999 |
| D\|e*c | h_exons_2 | - | mammalian_CNEs_1 | 38.692215 |
| D\|c*r | cnes85-90 | - | h_exons_rnd_3 | 38.665302 |

```
D|c*r |   amniotic CNEs      -  h_exons_rnd_2      |      38.476406
D|c*r |   cnes80-85          -  h_exons_rnd_3      |      38.355495
D|r*c |   h_exons_rnd_2      -  mammalian_CNEs_1   |      38.286705
D|c*r |   cnes75-80          -  h_exons_rnd_2      |      38.067001
D|c*r |   amniotic CNEs      -  h_exons_rnd_3      |      37.974216
D|r*c |   h_exons_rnd_2      -  mammalian_CNEs_2   |      37.957699
D|c*r |   cnes85-90          -  h_exons_rnd_1      |      37.957085
D|r*c |   h_exons_rnd_3      -  mammalian_CNEs_1   |      37.728687
D|e*c |   h_exons_2          -  ucnes95-100        |      37.669991
D|c*r |   cnes80-85          -  h_exons_rnd_1      |      37.634712
D|r*c |   h_exons_rnd_3      -  mammalian_CNEs_2   |      37.603893
D|c*r |   cnes75-80          -  h_exons_rnd_3      |      37.472778
D|c*r |   amniotic CNEs      -  h_exons_rnd_1      |      37.115795
D|r*c |   h_exons_rnd_1      -  mammalian_CNEs_1   |      36.845085
D|r*c |   h_exons_rnd_1      -  mammalian_CNEs_2   |      36.763111
D|c*r |   cnes75-80          -  h_exons_rnd_1      |      36.761585
D|c*e |   cnes90-95          -  h_exons_2          |      35.411110
D|e*c |   h_exons_1          -  mammalian_CNEs_2   |      35.272202
D|c*e |   amniotic CNEs      -  h_exons_2          |      34.189095
D|e*c |   h_exons_1          -  mammalian_CNEs_1   |      34.175388
D|e*c |   h_exons_3          -  mammalian_CNEs_2   |      33.987717
D|e*c |   h_exons_3          -  ucnes95-100        |      33.754478
D|e*c |   h_exons_1          -  ucnes95-100        |      33.689598
D|e*c |   h_exons_3          -  mammalian_CNEs_1   |      33.099697
D|c*e |   cnes85-90          -  h_exons_2          |      32.856785
S|c*c |   mammalian_CNEs_2   -  ucnes95-100        |      32.820797 #01
D|c*e |   cnes90-95          -  h_exons_1          |      31.718088
D|c*e |   cnes90-95          -  h_exons_3          |      31.539389
D|c*e |   cnes80-85          -  h_exons_2          |      30.602392
D|c*e |   amniotic CNEs      -  h_exons_1          |      30.067120
S|c*c |   mammalian_CNEs_1   -  ucnes95-100        |      30.035610 #02
D|c*e |   amniotic CNEs      -  h_exons_3          |      29.682201
D|c*e |   cnes75-80          -  h_exons_2          |      29.559093
D|c*e |   cnes85-90          -  h_exons_1          |      29.205807
S|c*c |   cnes90-95          -  mammalian_CNEs_2   |      28.542288 #03
D|c*e |   cnes85-90          -  h_exons_3          |      28.429094
D|c*e |   cnes80-85          -  h_exons_1          |      26.537003
D|c*e |   cnes80-85          -  h_exons_3          |      26.052288
D|c*e |   cnes75-80          -  h_exons_1          |      25.659115
S|c*c |   cnes90-95          -  mammalian_CNEs_1   |      25.623899 #04
D|c*e |   cnes75-80          -  h_exons_3          |      24.751223
=================================================================
S|c*c |   cnes85-90          -  mammalian_CNEs_2   |      24.169001
S|c*c |   cnes85-90          -  mammalian_CNEs_1   |      21.880989
S|c*c |   cnes80-85          -  mammalian_CNEs_2   |      21.535002
S|c*c |   cnes75-80          -  ucnes95-100        |      21.214901
S|c*c |   amniotic CNEs      -  mammalian_CNEs_2   |      21.209299
S|c*c |   cnes75-80          -  mammalian_CNEs_2   |      20.712696
```

| | | | | |
|---|---|---|---|---|
| S\|c*c | cnes80-85 | - | ucnes95-100 | 19.540997 |
| S\|c*c | cnes80-85 | - | mammalian_CNEs_1 | 19.188610 |
| S\|c*c | cnes75-80 | - | mammalian_CNEs_1 | 18.815699 |
| S\|c*c | amniotic CNEs | - | mammalian_CNEs_1 | 18.438694 |
| S\|c*c | cnes85-90 | - | ucnes95-100 | 17.853392 |
| S\|c*c | cnes75-80 | - | cnes90-95 | 17.261999 |
| S\|c*c | cnes80-85 | - | cnes90-95 | 16.243702 |
| S\|c*c | amniotic CNEs | - | ucnes95-100 | 15.607301 |
| S\|c*c | cnes85-90 | - | cnes90-95 | 14.393503 |
| S\|c*c | cnes90-95 | - | ucnes95-100 | 14.156302 |
| S\|c*c | cnes75-80 | - | cnes85-90 | 13.440700 |
| S\|c*c | cnes80-85 | - | cnes85-90 | 13.176803 |
| S\|r*r | cnes85-90_rnd | - | cnes90-95_rnd | 12.966905 |
| S\|r*r | cnes90-95_rnd | - | ucnes95-100_rnd | 12.948005 |
| S\|c*c | amniotic CNEs | - | cnes75-80 | 12.625998 |
| S\|c*c | amniotic CNEs | - | cnes80-85 | 12.269102 |
| S\|r*r | cnes85-90_rnd | - | ucnes95-100_rnd | 11.852503 |
| S\|r*r | cnes80-85_rnd | - | cnes85-90_rnd | 11.286098 |
| S\|c*c | amniotic CNEs | - | cnes90-95 | 11.283600 |
| S\|r*r | cnes75-80_rnd | - | cnes85-90_rnd | 11.049200 |
| S\|r*r | cnes80-85_rnd | - | ucnes95-100_rnd | 10.964605 |
| S\|e*e | h_exons_2 | - | h_exons_3 | 10.828105 |
| S\|r*r | cnes75-80_rnd | - | cnes90-95_rnd | 10.658503 |
| S\|r*r | cnes80-85_rnd | - | cnes90-95_rnd | 10.518397 |
| S\|e*e | h_exons_1 | - | h_exons_2 | 10.413592 |
| S\|c*c | amniotic CNEs | - | cnes85-90 | 10.279699 |
| S\|r*r | cnes75-80_rnd | - | ucnes95-100_rnd | 10.225303 |
| S\|r*r | h_exons_rnd_1 | - | ucnes95-100_rnd | 10.180299 |
| S\|r*r | h_exons_rnd_3 | - | ucnes95-100_rnd | 10.160304 |
| S\|r*r | cnes85-90_rnd | - | h_exons_rnd_1 | 10.148006 |
| S\|c*c | cnes75-80 | - | cnes80-85 | 10.114497 |
| S\|r*r | amniotic_rnd | - | cnes90-95_rnd | 10.097201 |
| S\|r*r | h_exons_rnd_2 | - | ucnes95-100_rnd | 9.972904 |
| S\|r*r | cnes90-95_rnd | - | h_exons_rnd_3 | 9.885698 |
| S\|r*r | amniotic_rnd | - | cnes85-90_rnd | 9.766304 |
| S\|r*r | cnes90-95_rnd | - | h_exons_rnd_1 | 9.723505 |
| S\|r*r | mammalian_rnd_1 | - | ucnes95-100_rnd | 9.679800 |
| S\|r*r | cnes85-90_rnd | - | h_exons_rnd_3 | 9.594798 |
| S\|r*r | mammalian_rnd_2 | - | ucnes95-100_rnd | 9.592600 |
| S\|r*r | amniotic_rnd | - | ucnes95-100_rnd | 9.568996 |
| S\|r*r | cnes90-95_rnd | - | mammalian_rnd_2 | 9.365602 |
| S\|r*r | cnes85-90_rnd | - | mammalian_rnd_2 | 9.316502 |
| S\|r*r | cnes90-95_rnd | - | mammalian_rnd_1 | 9.289997 |
| S\|r*r | cnes90-95_rnd | - | h_exons_rnd_2 | 9.261698 |
| S\|r*r | cnes85-90_rnd | - | mammalian_rnd_1 | 9.256298 |
| S\|r*r | cnes85-90_rnd | - | h_exons_rnd_2 | 9.255400 |
| S\|r*r | cnes75-80_rnd | - | cnes80-85_rnd | 8.672301 |
| S\|r*r | amniotic_rnd | - | cnes75-80_rnd | 8.195905 |

| | | | | | |
|---|---|---|---|---|---|
| S\|r*r | cnes80-85_rnd | - | h_exons_rnd_1 | \| | 7.660295 |
| S\|r*r | cnes80-85_rnd | - | h_exons_rnd_2 | \| | 7.598302 |
| S\|c*c | mammalian_CNEs_1 | - | mammalian_CNEs_2 | \| | 7.581599 |
| S\|r*r | amniotic_rnd | - | cnes80-85_rnd | \| | 7.532602 |
| S\|r*r | cnes80-85_rnd | - | mammalian_rnd_1 | \| | 7.526799 |
| S\|r*r | cnes80-85_rnd | - | mammalian_rnd_2 | \| | 7.458999 |
| S\|r*r | cnes75-80_rnd | - | h_exons_rnd_2 | \| | 7.417603 |
| S\|r*r | cnes80-85_rnd | - | h_exons_rnd_3 | \| | 7.366900 |
| S\|r*r | cnes75-80_rnd | - | h_exons_rnd_3 | \| | 7.225802 |
| S\|r*r | cnes75-80_rnd | - | h_exons_rnd_1 | \| | 7.180402 |
| S\|r*r | cnes75-80_rnd | - | mammalian_rnd_2 | \| | 7.112700 |
| S\|r*r | cnes75-80_rnd | - | mammalian_rnd_1 | \| | 7.106102 |
| S\|r*r | amniotic_rnd | - | h_exons_rnd_1 | \| | 6.435901 |
| S\|r*r | amniotic_rnd | - | h_exons_rnd_3 | \| | 5.867300 |
| S\|r*r | h_exons_rnd_1 | - | mammalian_rnd_1 | \| | 5.861905 |
| S\|r*r | h_exons_rnd_1 | - | mammalian_rnd_2 | \| | 5.762501 |
| S\|r*r | amniotic_rnd | - | h_exons_rnd_2 | \| | 5.668299 |
| S\|e*e | h_exons_1 | - | h_exons_3 | \| | 5.455503 |
| S\|r*r | amniotic_rnd | - | mammalian_rnd_1 | \| | 5.414001 |
| S\|r*r | h_exons_rnd_3 | - | mammalian_rnd_1 | \| | 5.328101 |
| S\|r*r | amniotic_rnd | - | mammalian_rnd_2 | \| | 5.243602 |
| S\|r*r | h_exons_rnd_2 | - | mammalian_rnd_1 | \| | 5.129900 |
| S\|r*r | h_exons_rnd_2 | - | mammalian_rnd_2 | \| | 4.882299 |
| S\|r*r | h_exons_rnd_1 | - | h_exons_rnd_2 | \| | 4.810199 |
| S\|r*r | mammalian_rnd_1 | - | mammalian_rnd_2 | \| | 4.797000 |
| S\|r*r | h_exons_rnd_3 | - | mammalian_rnd_2 | \| | 4.611102 |
| S\|r*r | h_exons_rnd_2 | - | h_exons_rnd_3 | \| | 4.456001 |
| S\|r*r | h_exons_rnd_1 | - | h_exons_rnd_3 | \| | 4.440000 |

## Supplementary Figure S2

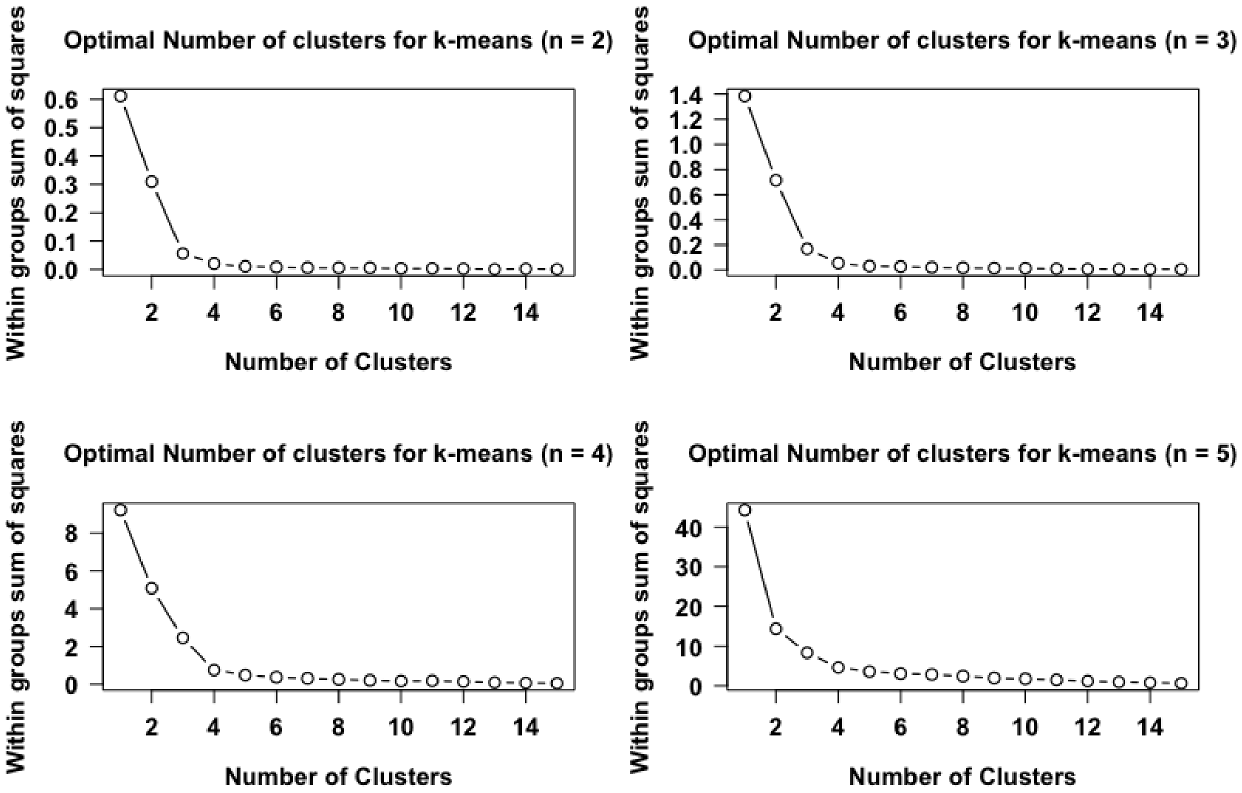


The “elbow” method to determine the optimal number of clusters for the k-means clustering of GGS feature vectors computed for CNE, exons and surrogate datasets, including mammalian CNEs. For the computation of GGS feature vectors, n can take values from 2 to 5.
